# Green Urea Synthesis from CO_2_ and Nitrogenous Small Molecules via Electrocatalysis and Photocatalysis

**DOI:** 10.1002/smsc.202500289

**Published:** 2025-07-14

**Authors:** Lizhen Liu, Lin Zhou, Longcheng Zhang, Hongwei Huang, Xin Zhao, Zhichuan J. Xu

**Affiliations:** ^1^ College of Resources and Environmental Sciences China Agricultural University Beijing 100193 China; ^2^ School of Materials Science and Engineering Nanyang Technological University Singapore 639798 Singapore; ^3^ School of Materials Science and Technology China University of Geosciences (Beijing) Beijing 100083 China; ^4^ Centre for Advanced Catalysis Science and Technology Nanyang Technological University Singapore 639798 Singapore

**Keywords:** catalytic sites, electrocatalysis, photocatalysis, urea detection, urea synthesis

## Abstract

Urea is an important and widely consumed compound in agriculture and pharmaceutical industries. Electrocatalytic and photocatalytic approaches enable green urea synthesis from CO_2_ and nitrogenous small molecules (N_2_, NO_3_^−^, NO_2_^−^, and NO), offering electron‐driven parallel routes that are alternative to Bosch–Meiser process with net‐zero emission potential. Although considerable efforts have achieved significant progress, current green urea synthesis is still far from the requirements of practical production due to sluggish reaction kinetics and low efficiency and selectivity of urea. Developing advanced catalysts and catalytic system is crucial for practical green urea synthesis. Therefore, in this review, the fundamentals of urea synthesis, covering the electrocatalytic and photocatalytic processes, thermodynamic and kinetic considerations, C—N coupling mechanism, and urea detection methods are introduced. Then, the pivotal role of the catalytic center in C—N coupling and recent breakthroughs in strategies for catalysts and reaction system design are summarized. Finally, potential directions for catalytic system optimization, standardization of product analysis, and scale‐up from laboratory to industry are proposed to guide future research on green urea synthesis.

## Graphical abstract

Electrocatalysis and photocatalysis offer electron‐driven parallel routes for green urea synthesis from CO_2_ and nitrogenous small molecules (N_2_, NO_3_^−^, NO_2_^−^, and NO), providing sustainable alternatives to Bosch–Meiser process. Gaining in‐depth insights into the underlying mechanisms, efficiency, selectivity, and techno‐economic analysis of electrocatalytic and photocatalytic urea synthesis is essential for the rational design and optimization of urea synthesis systems.

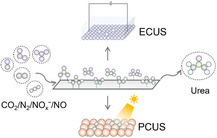

## 1 Introduction

**Figure 1:**
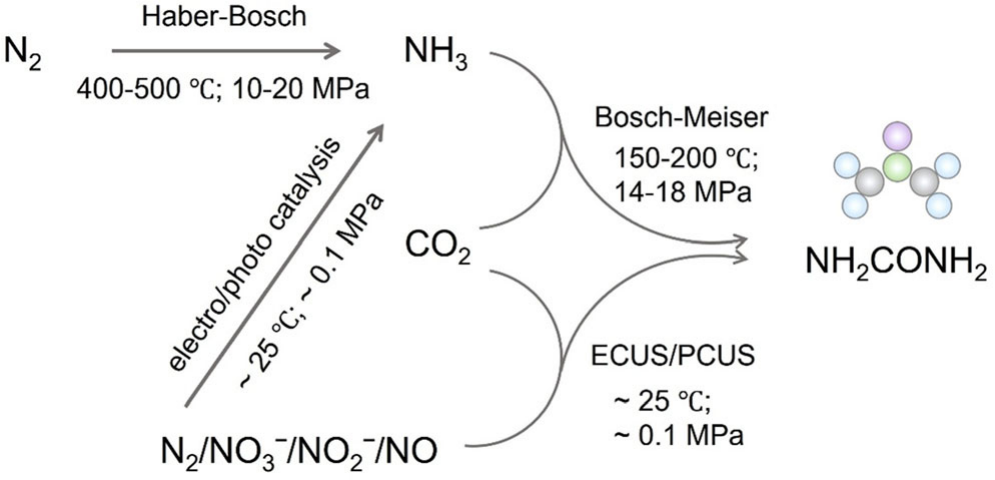
Schematic diagram of urea synthetic protocols in the conventional energy‐intensive pathway and ECUS/PCUS pathway.

Urea is an important organic compound in agricultural fertilizers to supply essential N‐containing nutrients for plant growth.^[^ ^1^ ^]^ More than 90% of urea is utilized in agriculture, with the remainder primarily employed in the synthesis of drugs, resins, and chemical products.^[^ ^2^ ^]^ The traditional method for urea synthesis is Bosch–Meiser process via NH_3_–CO_2_ system, which involves high temperature and pressure (150–200 °C; 14–18 MPa), and is accompanied by substantial energy consumption.^[^ ^3^, ^4^ ^]^ NH_3_ as the feedstock in urea production is mainly obtained from the Haber–Bosch process, which accounts for 2% of global energy consumption, further exacerbating the energy consumption of urea synthesis.^[^ ^5^ ^]^ Rapid industrialization requires sustainable energy and environmentally friendly technologies, which triggers the exploration of green urea synthesis.^[^ ^6^, ^7^ ^]^ Electrocatalytic urea synthesis (ECUS) and photocatalytic urea synthesis (PCUS) from CO_2_ and nitrogenous small molecules (such as N_2_, NO_3_^−^, NO_2_^−^, and NO) have attracted significant attention, offering sustainable pathways not only to produce eco‐friendly urea but also to reduce the flue gas pollutant and dependence on fossil energy (**Figure** **1**).

PCUS was first proposed by Hubbard et al. in 1975 via ultraviolet‐driven gas‐phase photocatalytic system using CO and NH_3_.^[^ ^8^ ^]^ Afterward, Kuwabata et al. employed size‐quantized TiO_2_ nanocrystals as photocatalysts to convert CO_2_ and NO_3_^−^ into urea in 1998.^[^ ^9^ ^]^ ECUS was first reported by Chen et al. in 2020 through coupling CO_2_ and N_2_ in H_2_O, sparking considerable interest in green urea synthesis.^[^ ^10^ ^]^ However, the processes of PCUS and ECUS still face challenges of CO_2_ and nitrogenous small molecules activation, and C—N coupling. The bond energy of C=O and N≡N are ∼800 and ∼945 kJ mol^−1^, respectively, which means a high energy barrier for CO_2_ and N_2_ activation.^[^ ^11^, ^12^, ^13^ ^]^ Using NO_3_^−^ and NO_2_^−^ as feedstocks can reduce the activation barrier of N‐reactants, but CO_2_ activation remains a significant challenge.^[^ ^14^ ^]^ Additionally, activation process is accompanied by CO_2_ reduction reaction (CO_2_RR) and nitrogenous small molecules reduction reaction (N_2_ reduction reaction: NRR; NO_3_^−^/NO_2_^−^/NO reduction reaction: NO_*x*_RR), leading to the formation of numerous byproducts that decrease the selectivity of urea.^[^ ^15^, ^16^ ^]^ The hydrogen evolution reaction (HER), commonly occurring in catalytic reduction processes, further affects the efficiency of ECUS and PCUS.^[^ ^17^ ^]^ Therefore, the high‐performance of urea synthesis requires catalysts with the following characteristics: 1) highly reactive catalytic sites; 2) superior activation ability for both C‐ and N‐reactants; 3) the match of specific intermediates with catalysts; and 4) the ability to suppress competitive reactions (CO_2_RR, N/NO_*x*_RR, and HER). In addition, the ECUS system, such as electrolyte, electric double‐layer effect, and pretreatment of reactants, is also worth exploring to improve the efficiency of urea production.^[^ ^18^, ^19^ ^]^ For the PCUS system, the light absorption capacity of catalysts and the type of aqueous solution also directly affect the efficiency of solar energy conversion and urea synthesis.^[^ ^20^ ^]^ Thereby, numerous efforts have been reported to develop the highly performance catalysts and catalytic system. For example, dual catalytic sites were designed to simultaneously activate CO_2_ and nitrogenous small molecules for favorable C—N coupling.^[^ ^21^, ^22^ ^]^ Tandem catalysts were constructed to control the reaction intermediates to suppress the byproducts.^[^ ^23^ ^]^ Pulsed potential was introduced to regulate the reaction microenvironment to enhance catalytic activity and selectivity.^[^ ^24^, ^25^ ^]^ These advances offer valuable insights for developing green urea synthesis. However, the efficiency is still far from the requirement of practical production. A deeper understanding of reaction mechanism and advanced reaction system should be provided to align with practical requirements. Therefore, a timely summary of updated progress and challenges in this immature field is essential to guide future research and development.

Several important reviews have addressed key aspects of urea synthesis, including design of catalysts,^[^ ^26^, ^27^ ^]^ mechanisms of C—N coupling,^[^ ^5^, ^28^, ^29^ ^]^ and guidelines for reliable urea detection,^[^ ^30^, ^31^ ^]^ providing valuable foundations. Building on this basis, exploring the mechanistic roles of specific catalytic sites may offer deeper insights into reaction pathways and product selectivity. In addition, catalytic system, including reaction medium, applied potentials, and feedstock pretreatment, plays a critical role in catalytic processes, yet remains underexplored. An integrated perspective that combines mechanistic understanding and system‐level considerations across ECUS and PCUS platforms is urgently needed. Therefore, an updated, in‐depth, and comprehensive review is of great significance and necessity.

Herein, we put an emphasis on recent breakthroughs in ECUS and PCUS, two electron‐driven approaches that offer green and parallel alternative to Bosch–Meiser process. First, fundamentals of urea synthesis are introduced, covering thermodynamic and kinetic considerations in urea synthesis, C—N coupling mechanism from CO_2_‐coupled N_2_/NO_3_^−^/NO_2_^−^/NO system, and urea detection methods, which provide in‐depth insight into reaction mechanism for urea synthesis and reliable product quantification methods. Then, the pivotal role of catalytic center in urea synthesis and the recent breakthroughs in strategies for catalysts and reaction system design are summarized, which promote the key steps for high activity and selectivity occurring in the urea synthesis, including the adsorption and activation of CO_2_ and nitrogenous small molecules, intermediate coupling, and inhibition of byproducts. Finally, perspectives for future development in ECUS and PCUS, including catalytic system design, standardization of product analysis, and transition from laboratory to industrial scale, are proposed to advance the green urea synthesis. This review aims to provide the latest updates in this field, with the goal of promoting green urea synthesis.

## 2 Fundamentals of Urea Synthesis

### 2.1 Bridged Mechanism of ECUS and PCUS

**Figure 2:**
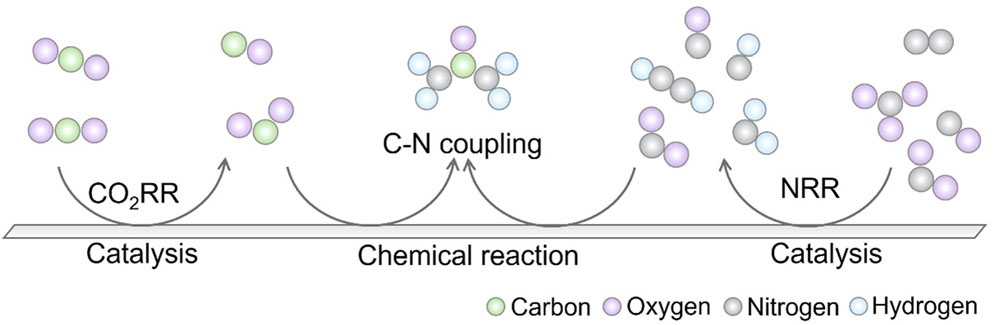
The scheme diagram of CO_2_ and nitrogenous small molecules activation and C—N coupling process in urea synthesis.

ECUS and PCUS possess a similar mechanism in the reaction process, which involves electron transfer to the catalytic sites, enabling the adsorption and activation of reactant molecules. The adsorbed reactants are converted into C‐ and N‐intermediates, followed by C—N coupling and subsequent protonation to form urea. The energy input relies on the energy carried by electrons in both ECUS and PCUS. In ECUS, electrons are supplied by an external electric circuit. In PCUS, electrons are mainly generated by the photocatalyst upon light excitation, relying primarily on solar energy conversion. Due to the limited energy carried by electrons, the activation of reactants and C—N coupling cannot be achieved in one step. Thus, various intermediates are formed during the urea synthesis, which results in a more complex process compared to the CO_2_RR and N/NO_x_RR. The intermediate species of CO_2_ contain *OCO, *CO, and *COOH, while the nitrogenous molecules are typically activated into *NN, *NHNH, *NO_2_, *NO, and *NH_2_, in which the C‐intermediate couples with N‐intermediate converted into various C—N intermediates, such as *NCON, *NHCONH, *CONO_2_, *OCNO, *CONH_2_, *OCONO_2_, and *OCONH_2_ (**Figure** **2**). The activation barriers and multiple intermediates of CO_2_ and N_2_/NO_3_^−^/NO_2_^−^/NO mainly determine the catalytic activity and the product selectivity from C—N coupling, respectively. The activation ability of catalytic centers, as well as the match between catalytic centers and specific intermediates significantly influence the performance of urea synthesis. Therefore, a thorough understanding of the reaction mechanism is crucial for achieving high reaction performance. Additionally, reliable urea detection method is prerequisite for accurately quantifying yield. Thus, urea detection methods are introduced to guide the urea analysis.

### 2.2 Thermodynamics and Dynamics of CO_2_ and N_2_/NO_3_^−^/NO_2_^−^/NO Activation

The CO_2_ and N_2_ molecules are inert chemical species, for which the C=O bond energy of ∼800 kJ mol^−1^ and N≡N bond energy of ∼945 kJ mol^−1^ are adverse to the activation process. The thermodynamic potential of CO_2_ activated to ·CO_2_^−^ is around −1.44 V (vs normal hydrogen electrode, pH = 0), which exceeds the conduction band (CB) position of most semiconductors in photocatalysis. The ionization potential of N_2_ is around 15.0 eV and its energy gap between the large highest occupied molecular orbital and the lowest unoccupied molecular orbital is ∼22.9 eV, which limits the electron transfer from catalysts to N_2_ for activation, especially in PCUS.^[^ ^32^ ^]^ In addition, the negative electron affinity of N_2_ is around −1.8 eV, which affects the N_2_ adsorption.^[^ ^33^ ^]^ In ECUS, this means that a larger input voltage is required to activate CO_2_ and N_2_ molecules compared to competing HER, which is a challenge for product selectivity. Kinetically, CO_2_ and N_2_ are nonpolar and linear symmetric molecules, the electron cloud density distribution of oxygen and nitrogen atoms is uniform, which leads to the stable structure of CO_2_ and N_2_ molecules, and slow activation process.^[^ ^34^ ^]^ Although the activation barriers and structural stability of NO_3_^−^ and NO_2_^−^ are relatively straightforward compared to CO_2_ and N_2_, the activation process involves multiple electrons and intermediates. The various reduction products from NO_*x*_RR are listed below^[^ ^35^, ^36^ ^]^

(1) $${\text{NO}}_{3}^{-}+2H^{+}+2e^{-}⇌{\text{NO}}_{2}^{-}+H_{2}O$$

(2) $${\text{NO}}_{3}^{-}+3H^{+}+2e^{-}⇌{\text{NHO}}_{2}+H_{2}O$$

(3) $${\text{NO}}_{3}^{-}+4H^{+}+3e^{-}⇌\text{NO}+2H_{2}O$$

(4) $${\text{NO}}_{3}^{-}+7H^{+}+6e^{-}⇌{\text{NH}}_{2}\text{OH}+2H_{2}O$$

(5) $${\text{NO}}_{3}^{-}+9H^{+}+8e^{-}⇌{\text{NH}}_{3}+3H_{2}O$$

(6) $$2{\text{NO}}_{3}^{-}+10H^{+}+8e^{-}⇌N_{2}O+5H_{2}O$$

(7) $$2{\text{NO}}_{3}^{-}+12H^{+}+10e^{-}⇌N_{2}+6H_{2}O$$

(8) $${\text{NO}}_{2}^{-}+2H^{+}+e^{-}⇌\text{NO}+H_{2}O$$

(9) $${\text{NO}}_{2}^{-}+8H^{+}+6e^{-}⇌{\text{NH}}_{4}^{+}+2H_{2}O$$

(10) $$2{\text{NO}}_{2}^{-}+12H^{+}+10e^{-}⇌N_{2}+6H_{2}O$$

These multielectron steps and intermediates render the C—N coupling pathway and its mechanism significantly more complex, which undermines product selectivity. Furthermore, compared with NO_3_^−^ and NO_2_^−^, the activation of NO involves fewer steps, which theoretically makes it more favorable for C—N coupling with *CO or *COOH intermediates. However, NO is prone to over‐reduction to NH_3_ or N_2_. In electrocatalytic systems, NO as an intermediate can readily dissociate into *N or *O species, which may lead to catalyst poisoning or deactivation. Therefore, a deeper understanding of the catalytic processes and reaction mechanisms of different N‐feedstocks is crucial for the rational design of efficient catalytic systems.

### 2.3 Mechanism of C—N Coupling

#### 2.3.1 C—N Coupling by CO_2_ and N_2_ as Feedstocks

**Figure 3:**
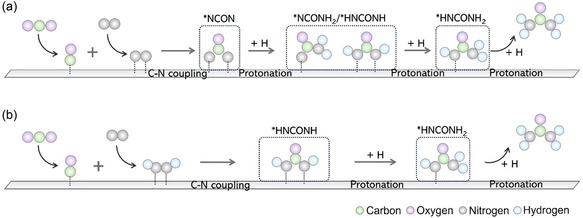
The mechanism diagram of CO_2_ activation, N_2_ activation, C—N coupling, and protonation process between CO_2_ and N_2_ intermediates: a) *CO + *NN and b) *CO + *HNNH pathway.

N_2_ is an earth‐abundant gas, and its direct use as a nitrogen source represents an environmentally friendly approach for urea synthesis. Moreover, its simple composition helps ensure high selectivity in urea synthesis. However, the strong inertness of N_2_ molecules poses a challenge, requiring catalysts with high activation ability. In general, the C—N coupling reaction between CO_2_ and N_2_ to urea includes four main steps (**Figure** **3**): 1) CO_2_ and N_2_ adsorption on the surface of catalysts; 2) CO_2_ and N_2_ are activated to *CO and *NN or *NHNH on the active sites, respectively; 3) the *CO and *NN/*NHNH couple to *NCON/*NHCONH; and 4) the *NCON or *NHCONH protonates to urea. CO_2_ and N_2_ underwent activation process to form adsorbed *CO and *NN species on the PdCu alloy surface, followed by C—N coupling to form *NCON intermediates, finally producing urea via further protonation.^[^ ^10^ ^]^ The C—N coupling between *NN and *CO on PdCu alloy surface is a thermodynamically favorable reaction, facilitating the formation of *NCON as a key intermediate for urea production. Consequently, the rate of urea formation reached 3.36 mmol g^−1^ h^−1^ with corresponding Faradaic efficiency (FE) of 8.92% at −0.4 V versus reversible hydrogen electrode (RHE). While on the MoP‐(101) surface, the N_2_ was activated and hydrogenated to *HNNH and then coupled with *CO to form *NHCONH, resulting in urea formation rate of 12.4 μg mg_cat._^−1^ h^−1^ and the FE of 36.5% at −0.35 V versus RHE.^[^ ^37^ ^]^ In this reaction, the subsequent hydrogenation of *NHCONH is an endothermic process, which requires the catalysts with strong protonation ability while preventing hydrogenation byproducts from CO_2_ and N_2_. This is a balance in urea synthesis.

#### 2.3.2 C—N Coupling by CO_2_ and NO_3_^−^/NO_2_^−^/NO as Feedstocks

**Figure 4:**
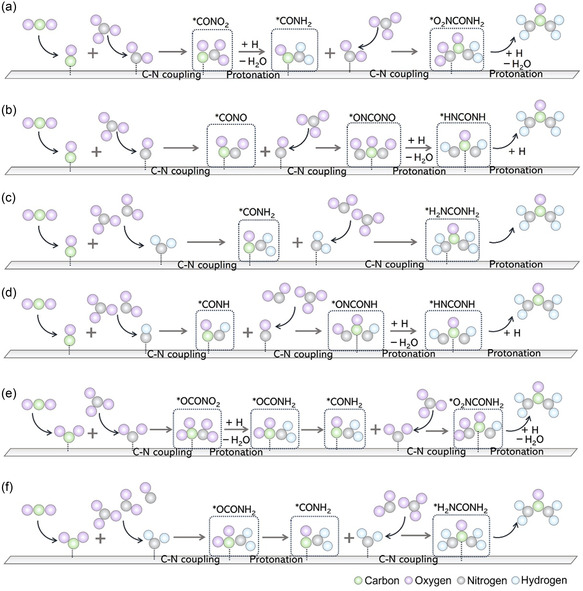
The mechanism diagram of CO_2_ activation, NO_3_^−^/NO_2_^−^/NO activation, C—N coupling, and protonation process between CO_2_ and NO_3_^−^/NO_2_^−^/NO intermediates: a) *CO + *NO_2_, b) *CO + *NO, c) *CO + *NH_2_, d) *CO + *NH, e) *CO_2_ + *NO_2_, and f) *CO + *NH_2_ pathway.

NO_3_^−^, NO_2_^−^, and NO are common pollutants in industrial emissions from the electroplating industry. Utilizing NO_3_^−^/NO_2_^−^/NO as the feedstocks for urea synthesis represents a promising approach to converting pollutants into high‐value products. NO_3_^−^/NO_2_^−^/NO offers the advantage of feedstock transportation compared to N_2_ and NH_3_, facilitating the practical application of green urea synthesis. Moreover, NO_3_^−^/NO_2_^−^/NO is more readily activated than N_2_, facilitating lower energy barriers for subsequent reactions. However, the activation process of NO_3_^−^/NO_2_^−^/NO involves more steps compared to N_2_, with various intermediates.^[^ ^38^, ^39^ ^]^ The adsorption intermediates of NO_3_^−^/NO_2_^−^/NO and CO_2_ include *NO_3_, *NO_2_, *NO, *NH, and *NH_2_, as well as *CO_2_ and *CO, respectively, where six types of intermediates have been reported in the first step of C—N coupling, including *CONO_2_, *CONO, *CONH_2_, *CONH, *OCONO_2_, and *OCONH_2_ (**Figure** **4**). These intermediates of NO_3_^−^/NO_2_^−^/NO and CO_2_ face proton‐coupling electron‐transfer processes and chemical steps (C—N coupling).^[^ ^40^, ^41^ ^]^ Thus, the high product selectivity remains a critical challenge to be addressed. Wei et al. proposed that utilizing oxygen vacancy (O_v_) mediated selectivity of C—N coupling with protonation, where the O_v_ can suppress the *NO hydrogenation while promote C—N coupling of *CO and *NO on CeO_2_ surface.^[^ ^42^ ^]^ The O_v_ enabled the coupling reaction of *CO and *NO to be more favorable than that of *H and *NO. Leverett et al. tuned the coordination structure of active sites to control the selectivity of urea synthesis.^[^ ^43^ ^]^ The Cu–N_4_ sites exhibited higher intrinsic activity toward the CO_2_RR, while Cu–N_4−*x*_–C_*x*_ sites showed activity toward the NO_*x*_RR. In this work, a catalyst with both appropriate CO_2_RR and NO_*x*_RR activity, Cu–N_3_–C_1_, was reported for C—N coupling, which achieved a FE of 28% for urea production at −0.9 V versus RHE. Thus, regulation of CO_2_RR and N/NO_*x*_RR processes is crucial, where the active sites should not only exhibit activity for CO_2_RR, N/NO_*x*_RR, and C—N coupling but also effectively suppress the byproducts from CO_2_RR and N/NO_*x*_RR.

### 2.4 Methods for Urea Detection

Currently, the commonly employed urea detection techniques include nuclear magnetic resonance (NMR) spectroscopy, liquid chromatography–mass spectrometry (LC–MS), and ultraviolet–visible (UV–vis) spectroscopy [including diacetyl monoxime (DAMO) and urease decomposition (UDC) methods].

NMR method is employed to analyze and quantify urea content by leveraging the distinctive chemical environment of hydrogen atoms in urea molecules, which produces characteristic chemical shift signals in the NMR spectrum.^[^ ^44^ ^]^ This method involves mixing the test solution with a deuterated reagent and an internal standard, transferring the mixture into an NMR tube, and analyzing it using a Fourier transform nuclear magnetic resonance spectrometer. The urea concentration is determined by comparing the integrated area of the characteristic peak of the internal standard with that of urea. The NMR analysis requires no pretreatment, and the determination is unaffected by reaction byproducts or metal ions, offering advantages such as minimal interference, simplicity, and high accuracy. Additionally, NMR method can detect both nonisotope‐labeled urea and ^15^N‐labeled urea.^[^ ^45^ ^]^ However, the quality of the data largely depends on the proficiency of the equipment and the expertise of the operators, while the method is associated with high costs.

LC‐MS method combines the superior separation capability of liquid chromatography with the high sensitivity and selective detection of mass spectrometry, allowing for efficient separation and analysis based on the chemical composition and physical properties of urea molecules. The m/z of urea is 61. If one or N atom in urea is replaced by ^15^N, the m/z of urea will shift to 62 or 63. In quantitative analysis, LC‐MS determines urea concentration based on the characteristic ion signals corresponding to each isotopic variant, allowing for high accuracy and specificity. This method offers greater convenience, simpler operation, and minimal interference from extraneous substances compared to NMR method. Although the method requires careful optimization of the mobile phase, flow rate, and detection parameters, while being associated with high operational costs, LC‐MS remains the preferred technique for quantifying urea in ppm‐level samples, as widely recommended in reported research works.^[^ ^46^, ^47^ ^]^

DAMO method employs a colorimetric reaction using UV–vis spectroscopy, in which urea hydrolyzes in the presence of DAMO under acidic conditions and Fe^3+^ as a catalyst. This reaction produces a pink complex, and the absorbance of this complex at 525 nm is then used to quantify urea. In the experiment, two chromogenic agents (named as A and B) are required. Reagent A is prepared by mixing 100 mL of concentrated phosphoric acid, 300 mL of concentrated sulfuric acid, and 600 mL of ultrapure water, followed by the addition of 0.1 g of anhydrous ferric chloride. Reagent B is prepared by dissolving 5 g of diacetyl monoxime and 0.1 g of thiosemicarbazide in 100 mL of ultrapure water. After the reaction, 1 mL of the reaction solution is collected from the reaction cell and mixed with 2 mL of reagent A and 1 mL of reagent B. The mixture is then heated at 100 °C for 15 min. After cooling the solution to room temperature, the absorbance is measured by UV–vis spectroscopy. The DAMO method offers several advantages, including low cost, simple operation, fast detection, high sensitivity, and good reproducibility, making it the most commonly used method for urea detection.^[^ ^48^ ^]^ However, its accuracy can be compromised by interferences from substances, such as NO_2_^−^ (>10 mM), reducing agents (such as thiourea, thiosulfate), high‐concentration electrolytes, and metal cations. High concentrations of NO_2_^−^ react with the chromogenic agent, leading to product fading, while low concentrations of NO_2_^−^ may result in false positives.^[^ ^49^, ^50^ ^]^ Consequently, the DAMO method is unsuitable for reaction system containing NO_2_^−^ or those with significant NO_2_^−^ byproducts. Additionally, an acidic environment is required for color development, which may lead to the decomposition of urea and affect the accurate quantification of urea. Other reducing agents, such as thiourea and thiosulfate, also interfere with the accuracy of the DAMO method. Therefore, the use of the DAMO method requires careful control of the pH during color development and the avoidance of interference from NO_2_^−^ and other potentially disruptive factors.

UDC method utilizes urease to break down urea into ammonia and carbon dioxide, followed by the measurement of ammonia content with additional equipment to quantify the urea. This method is simple and highly specific, with relatively straightforward experimental procedures that typically do not require complex instruments or equipment. It has been a common method for urea detection since 1995.^[^ ^46^ ^]^ However, it is limited by the activity of urease and exhibits low sensitivity, making it less suitable for detecting urea at ppm‐level concentrations in ECUS and PCUS systems.^[^ ^51^ ^]^

**Figure 5:**
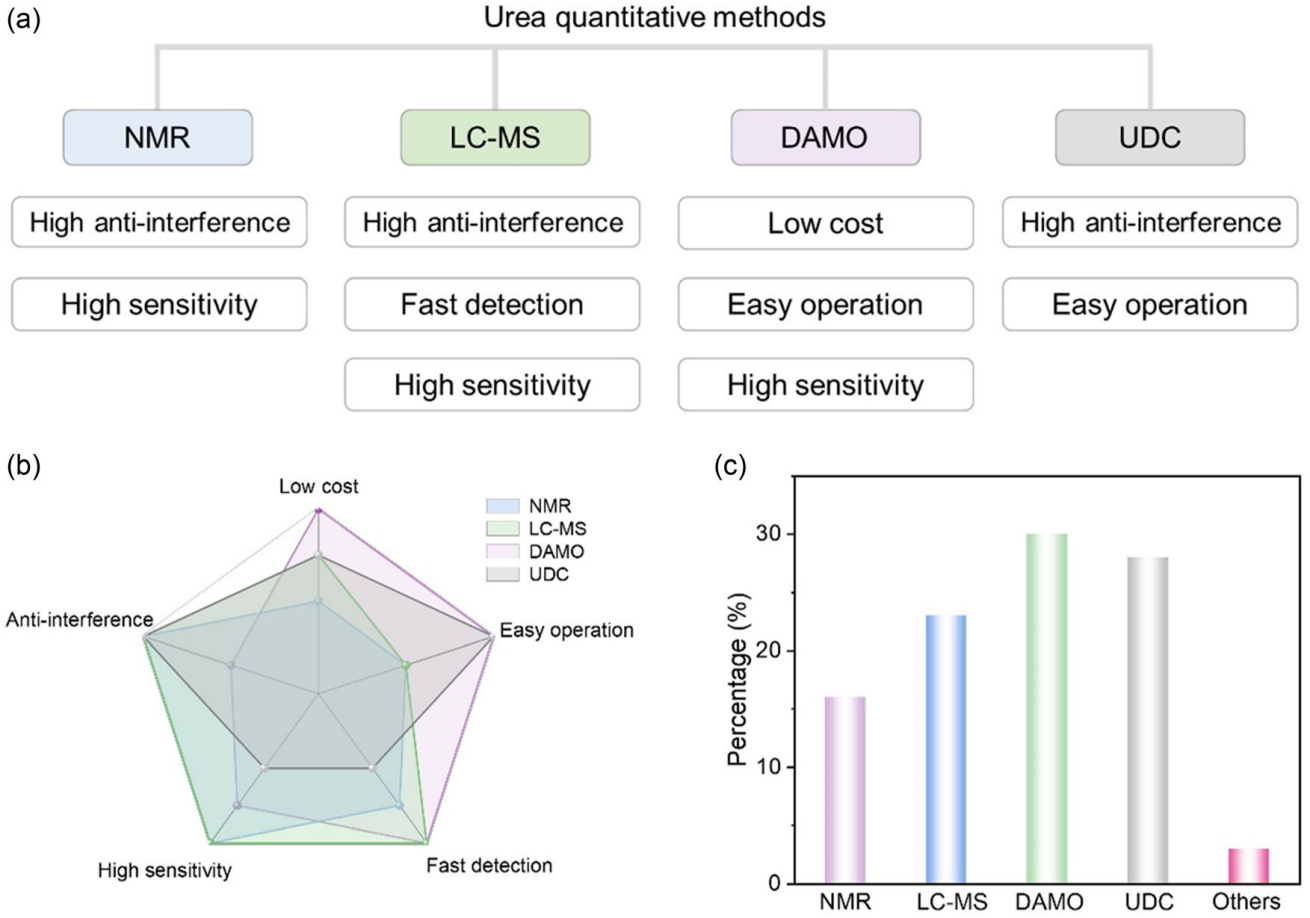
a) The advantages, b) the comparison, and c) the usage frequency of NMR, LC–MS, DAMO, and UDC methods in current ECUS and GCUS systems.

These four detection methods present distinct advantages and disadvantages. Therefore, we have summarized the sensitivity, interfering factors, advantages, and usage frequency of each method to provide a comprehensive reference for urea detection in various experimental settings (**Figure** **5**).^[^ ^47^, ^48^ ^]^

## 3 Electrocatalytic Urea Synthesis

### 3.1 Electrocatalysts

**Figure 6:**
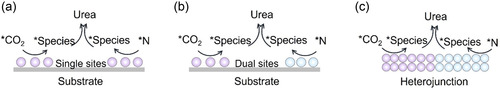
The schematic diagram of urea synthesis on a) catalysts with single‐atom sites, b) catalysts with dual‐atom sites, and c) heterostructural catalysts (*N: adsorbed nitrogenous molecules).

The electrocatalysis requires that catalysts provide sufficient and efficient catalytic centers for CO_2_ and nitrogenous molecules activation. To meet these demands, the reported works mainly focused on exploring various catalytic sites, including single‐atom and dual‐atom sites to overcome the activation barriers associated with these reactants.^[^ ^52^, ^53^ ^]^ These unique catalytic configurations demonstrate exceptional performance on different catalysts. Furthermore, the heterojunction structure is also proposed to provide distinct catalytic regions, facilitating the activation process of CO_2_ and nitrogenous molecules. Therefore, in this section, we primarily summarize catalysts featuring single, dual sites, and heterostructure (**Figure** **6**), to elucidate the design principles underlying their high catalytic performance.

#### 3.1.1 Electrocatalysts with Single Site

**Figure 7:**
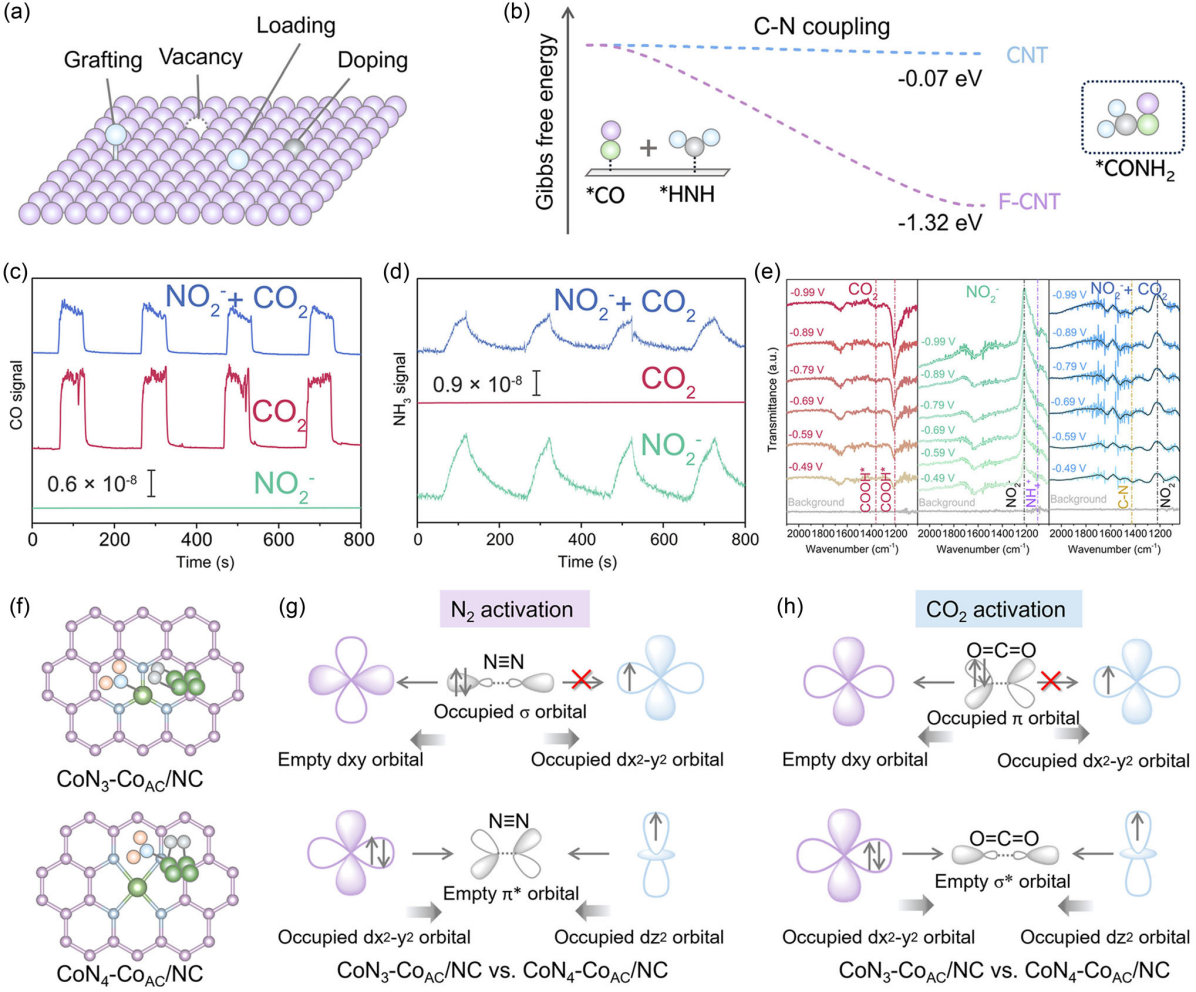
a) Schematic illustration of different types of single site. b) Potential energy diagrams of C—N coupling reaction, on CNT and F‐doped CNT active sites. Reproduced with permission.^[^ ^54^ ^]^ Copyright 2022, Elsevier. Online DEMS spectra of c) CO and d) NH_3_ signals over ZnO–V; e) in situ attenuated total reflectance fourier transform infrared spectroscopy curves of ZnO–V under CO_2_, NaNO_2_, and both. Reproduced with permission.^[^ ^55^ ^]^ Copyright 2021, Elsevier. f) Simplified schematic of N_2_ and CO_2_ molecules adsorbed on CoN_3_–Co_AC_/NC and CoN_4_–Co_AC_/NC; schematic illustration of charge “donation‐acceptance” process between g) CoN_3_–Co_AC_/NC or CoN_4_–Co_AC_/NC and CO_2_ molecule; h) CoN_3_–Co_AC_/NC or CoN_4_–Co_AC_/NC and N_2_ molecule. Reproduced with permission.^[^ ^56^ ^]^ Copyright 2025, Elsevier.

The reported single site involved in urea synthesis primarily includes Ti, Co (including Co^0^, high‐spin state Co^3+^, and intermediate‐spin state Co^4+^), Cu (including Cu^0^, Cu^1+^, Cu^δ+^, and Cu^2+^), Zn, Mo, In, Pt, Au, Bi, C, N, and O_v_ sites (**Figure** **7**a). The activation of CO_2_ and nitrogenous molecules into adsorbed species both occurs at the reduction site, enabling the catalytic potential of catalysts with single site.

F‐doped carbon nanotubes as metal‐free electrocatalyst were prepared for urea synthesis, which increased the interlayer spacing of the nanotubes, offering more catalytic sites.^[^ ^54^ ^]^ The F‐doped C sites enhanced activity for CO_2_ activation to *COOH and NO_3_^−^ reduction to *NH_2_ compared to undoped carbon sites (Figure 7b). The modified C sites demonstrated high‐catalytic performance in both CO_2_RR and NO_*x*_RR, achieving a urea yield rate of 6.36 mmol h^−^^1^ g_cat._^−^^1^ with a FE of 18.0% at −0.65 V versus RHE. This yield is 3.8 times higher than that of the pristine carbon nanotube (CNT) sample. The introduction of O_v_ can increase the electrochemically active surface area (ECSA) of catalysts to provide more active sites. Meng et al. introduced O_v_ sites onto ZnO porous nanosheets (ZnO‐V), where the O_v_ are uniformly distributed across the ZnO‐V surface, creating an amorphous layer with abundant O_v_ catalytic sites.^[^ ^55^ ^]^ The O_v_ efficiently increased the ECSA and decreased interfacial charge transfer resistance for ZnO‐V, and realized the CO_2_ and NO_2_^−^ coadsorption to accelerate urea formation. Afterward, online differential electrochemical mass spectrometry (DEMS) recorded the signal of CO and NH_3_ under CO_2_ and NaNO_2_, which indicated the signal intensities of CO and NH_3_ decreased under CO_2_ + NaNO_2_ (Figure 7c,d). Combined with the in situ FTIR results, it indicated that the adsorbed CO_2_ and NO_3_^−^ were converted into *COOH and *NH_2_ intermediates for C—N coupling (Figure 7e).

Transition metals exhibit superior electron transfer activity compared to nonmetallic sites, demonstrating superior activity in coactivation of CO_2_ and N_2_/NO_*x*_^−^. The empty *d*_*xy*_ orbitals and fully occupied $d_{x-y}^{2}$ orbitals of Co sites create an enabling environment for coactivation of N_2_ and CO_2_ (Figure 7f).^[^ ^56^ ^]^ The Co coordination environment provided unique electron delocalization effects and further prompted the orbital spin state of Co sites evolved from 3*d* ^7^4*s* ^1^ to 3*d* ^8^4*s* ^0^. The interaction between the σ* orbital of CO_2_ and occupied $d_{x-y}^{2}$ orbital of Co generated the apparent bonding state ($d_{x-y}^{2}$–σ*) for donating electrons from fully‐filled $d_{x-y}^{2}$ orbital to empty σ* orbital (Figure 7 g). The similar electron transfer has been observed in activating N_2_ molecule (Figure 7h). The targeted electron transfer can simultaneously facilitate CO_2_ and N_2_ activation, which exhibited the record highest urea yield rate of 20.83 mmol h^−1^ g^−1^ with FE of 23.73% at −0.4 V versus RHE at that time. However, transition metals tend to exhibit structural instability during activation processes due to their variable valence states, leading to challenges in long‐term catalytic activity. Fortunately, prior works demonstrated that regulating structural reconstruction of transition metals can enhance their stability. For example, the Cu single atom underwent structural reconstruction, transforming into Cu clusters, which increased the stability of Cu catalytic sites and served as the real active species in urea synthesis.^[^ ^57^ ^]^ Control of atom‐scale spacing (*d*_s_) for Cu can enhance the stability of transition states on the top Cu surface.^[^ ^58^ ^]^ The *d*_s_ influenced the transferred electron density from the upper surface to substrates, which can maintain the structural stability and lower the energy barrier for C—N coupling to provide a long‐term catalytic stability.

Different facets exhibit distinct catalytic performance in urea synthesis. The Cu_2_O (100) facet exhibited higher urea activity and selectivity than the (110) facet, where the Cu^1+^ and Cu^0^ can synergistically reduce the energy barriers of hydrogenated *NO on the (100) facet to facilitate C—N coupling between *CO and *NH intermediates.^[^ ^59^ ^]^ The (100) facet of In(OH)_3_ exhibited superior performance compared to the (110) facet.^[^ ^60^ ^]^ On the (100) facet, *NO_2_ and *CO_2_ intermediates preferentially coupled directly at an early stage, whereas on the (110) facet, adsorbed *CO_2_ required activation to *CO before coupling with *NO_2_. This difference in reaction pathways contributes to the facet‐dependent catalytic activity of In(OH)_3_. The different facets exhibit distinct catalytic behavior, particularly in intermediate conversion pathways and activation energy barriers, which reflects the significance of facet engineering in catalyst design.^[^ ^61^ ^]^

Although single catalytic site can simultaneously activate CO and N_2_, the catalytic performance is still limited due to the inherent limitations of the single active center. Its coordination environment or electronic configuration is often tailored to meet the activity requirements of both N/NO_*x*_RR and CO_2_RR.^[^ ^21^ ^]^ Therefore, a one‐to‐one catalytic mode has been proposed to maximize the catalytic performance, leading to the development of dual‐site catalysts.

#### 3.1.2 Electrocatalysts with Dual Sites

Dual sites on catalyst can be designed with separate catalytic sites for N/NO_*x*_RR and CO_2_RR, ensuring spatial separation of their respective catalytic environments.^[^ ^62^ ^]^ This design enables the optimization of catalytic centers for each reaction, thereby maximizing their individual catalytic potential. Moreover, synergistic interactions between two active sites can further enhance the efficiency of urea synthesis. The reported dual‐site combinations include Cu–M (M = Ti, Co, Ni, Mo, Zn, Pt, Au, Ru, Pd_1_, Rh_1_, Si, Bi, W, N, O_v_), Cu_1_–Cu_4_ (single Cu‐cluster Cu), Fe–Fe^2+^/Fe^3+^, Fe–Ni, Fe–N, Co–Mo, Co–Ru, Zn–Mn, Pd–Au, Pd–In, Pd–Zn, Sb–Bi, Ru–O_v_, Ti–C, Lewis pairs (In–OH, Ni–OH), etc., which involve metal‐metal, metal‐nonmetal, and Lewis pair, fully leveraging the strengths of each catalytic center.

**Figure 8:**
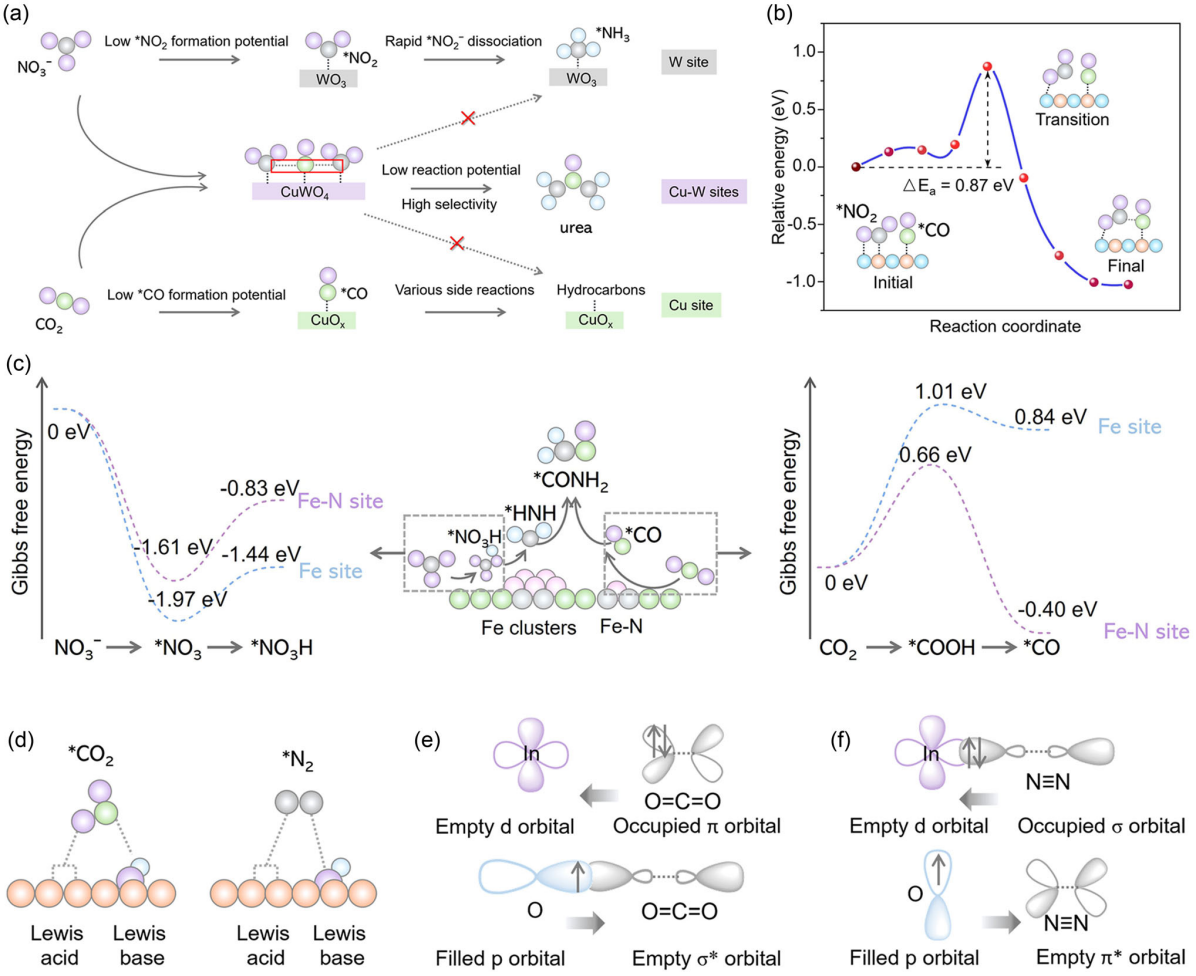
a) The stabilization of the activated *CO and *NO_2_ intermediates at Cu and W bimetallic sites may play an important role in the high efficiency of C—N coupling; b) mechanism of C—N coupling between *CO and *NO_2_. The initial, transition, and final states during the *CONO_2_ formation are presented. Gray, blue, red, orange, cyan, and yellow balls represent C, N, lattice O, Cu, W, and adsorbate O, respectively. Reproduced with permission.^[^ ^65^ ^]^ Copyright 2023, Springer Nature. c) Structural schematic diagram of F–N site and Fe site, and the corresponding Gibbs free energies for NO_3_^−^ and CO_2_ activation. Reproduced with permission.^[^ ^66^ ^]^ Copyright 2024, Wiley‐VCH. d) Schematic illustration of N_2_ and CO_2_ molecules adsorbed on independent LA site, LB site, and FLP site; schematic illustration of charge donation‐acceptance process between FLP and e) CO_2_ molecule, and f) N_2_ molecule. Reproduced with permission.^[^ ^68^ ^]^ Copyright 2022, Elsevier.

The dispersed MN_3_–M′N_4_ (M and M′ represent metal atom) moiety across 26 homonuclear and 650 heteronuclear di‐metal systems were investigated to analyze the pivotal role of interaction and configuration of metal catalytic center in urea synthesis. 205 stable configurations were identified based on the principle of lowest energy and validated through ab initio molecular dynamics simulations.^[^ ^63^ ^]^ Considering three potential reaction pathways, (*NCON, *CO, and *OCOH) for urea synthesis, a five‐step high‐throughput screening approach was developed to identify catalysts with superior activity, while a complementary five‐aspect screening strategy was proposed to evaluate catalytic selectivity. This work indicated three favorite M–M′ (Fe–Os, Co–Os, and Ru–Co) electrocatalysts for urea production, and the RuN_3_–CoN_4_ combination was identified as the most favorable candidate with an exceptionally low limiting potential of −0.80 V for urea synthesis, which demonstrated the advantage of dual catalytic sites in urea synthesis. However, the spatially isolated dual catalytic sites tend to generate byproducts when encountering hindered C—N coupling processes. Tandem catalysts enable stepwise reaction pathways to suppress the byproducts. A tandem catalyst featuring Mo–Co dual sites showed that Mo sites facilitated NO_3_^−^ reduction to the *NH_2_ intermediate, while Co sites promoted CO_2_ activation to *CO, thereby synergistically enhancing C—N coupling.^[^ ^64^ ^]^ The efficient C—N coupling is attributed to a tandem mechanism, in which the *NH_2_ and *CO intermediates generated at the Mo and Co active sites stabilized the formation of the *CONH_2_ intermediate to promote urea production, offering an effective strategy for the tandem catalyst design. Typically, different catalytic centers exhibit varying adsorption and activation capabilities toward different reactants. Therefore, the synergistic interaction between two catalytic centers can lead to superior catalytic performance. WO_3_ can convert NO_3_^−^ to NH_3_ and CuO_*x*_ can produce hydrocarbons from CO_2_. However, CuWO_4_ is limited to produce NH_3_ and hydrocarbons, yielding only urea when NO_3_^−^ and CO_2_ are used as reactants. This results from the combined advantages of W and Cu catalytic centers, which effectively activate NO_3_^−^ and CO_2_, enhance C—N coupling, and suppress side reactions (**Figure** **8**a).^[^ ^65^ ^]^ The high‐valence W center played a key role in stabilizing *NO_2_, lowering the reaction barrier, and increasing selectivity. Prior to the construction of Cu–W dual catalytic sites, NO_3_^−^ adsorbed on WO_3_ predominantly underwent conversion to ammonia, accompanied by a high‐activation energy barrier, while CO_2_ adsorbed on CuO_*x*_ favored hydrocarbon formation with low product selectivity. The introduction of alternating Cu–W bimetallic sites significantly reduced the reaction potential and enhanced selectivity for urea synthesis, effectively optimizing the synergistic catalytic function of both active sites (Figure 8b). The coordination environments or configuration of metal centers can also catalyze the CO_2_ and nitrogenous molecules separately in urea synthesis. Fe clusters (FeNC) and Fe_1_N_4_ configurations prefer NO_3_^−^ and CO_2_ adsorption, respectively (Figure 8c).^[^ ^66^ ^]^ The FeNC–Fe_1_N_4_/C catalyst with coexistence of Fe_1_N_4_ and FeNC demonstrated synergistic interaction of Fe_1_N_4_ and FeNC in urea production, where the urea production of FeNC–Fe_1_N_4_/C is higher than that of γ‐Fe_2_O_3_/C, FeNC/C and Fe_1_N_4_/C. The optimized metal coordination environment is crucial for improving selectivity and efficiency in urea synthesis. This approach promotes the investigation of metal site coordination and valence states, enabling a deeper understanding of their influence on catalytic pathways. Such insights provide valuable guidance for the rational design of high‐performance catalysts.

In catalytic reactions, NO_3_^−^/NO_2_^−^ exhibits Lewis basicity (electron donor) and can coordinate with Lewis acidic metal centers, while CO_2_ molecule acts as a Lewis acid (electron acceptor) and interacts with Lewis basic active sites. In addition, although N_2_ exhibits limited electron‐donating capability, many transition metals or metal oxides feature unoccupied orbitals or vacant *d*‐orbitals that are capable of interacting with the molecular orbitals of N_2_ through *π* orbital overlap, enabling N_2_ adsorption. Therefore, the design of Lewis acid–base sites has also been applied to catalyze N_2_/NO_3_^−^/NO_2_^−^ and CO_2_, thereby promoting urea synthesis. Yuan et al. proposed a unique frustrated Lewis pairs (FLPs) in the flower‐like nickel borate structure, Ni_3_(BO_3_)_2_, where surface hydroxyl groups and adjacent Ni sites act as Lewis base and acid, respectively.^[^ ^67^ ^]^ These FLPs enable the activation of inert CO_2_ and N_2_ through orbital interactions. Specifically, the bonding and antibonding orbitals of CO_2_ and N_2_ interact with the nonbonding orbitals of the Lewis base and the empty orbitals of the Lewis acid, facilitating molecular activation by the FLPs. This catalyst achieved the record‐high urea yield rate of 9.70 mmol h^−1^ g_cat._^−1^ and a FE of 20.36% at −0.5 V versus RHE at that time. To confirm that the urea originated from the electrocatalytic N_2_ and CO_2_ coreduction, a series of control experiments were conducted, including electrolysis in sole CO_2_‐saturated solution, with a bare carbon cloth electrode, or mixed gas (N_2_ + CO_2_)‐saturated electrolyte with an open circuit potential. In all cases, no obvious urea formation was detected. In addition, isotopic labeling experiments were carried out. When using ^15^N_2_ and CO_2_ as feedstocks, the signal corresponding to CO(^15^NH_2_)_2_ was detected in the ^1^H NMR spectrum, confirming that the urea was generated via the electrocatalytic process. This work demonstrated that the Lewis acidic sites can also interact with N_2_ molecules for NRR. Furthermore, the rice‐like InOOH nanoparticles, featuring both Lewis acidic In sites and electron‐rich Lewis basic In–OH sites (Figure 8d), demonstrated exceptional performance in urea synthesis.^[^ ^68^ ^]^ This was achieved through targeted chemisorption of N_2_ and CO_2_ molecules via electronic interactions, which effectively promoted the NRR and CO_2_RR processes, respectively. The FLPs activated the N≡N bond in N_2_ and the C=O bond in CO_2_ molecules through the empty orbitals of In sites and the nonbonding orbitals of In–OH sites, respectively, thereby promoting the formation of *N=N and *CO intermediates (Figure 8e,f). These intermediates then coupled to form the *NCON intermediate, which subsequently underwent the hydrogenation to ultimately produce urea. The spatially distributed FLPs provide electron‐rich and electron‐deficient sites as well as empty and nonbonding orbitals, which are highly favorable for the adsorption and activation of N_2_ and CO_2_ molecules, and the urea synthesis. The FLPs provide an effective reference for activating inert gas molecules in heterogeneous catalysis.

The design of dual catalytic sites can effectively enhance urea synthesis performance by creating spatially separated activation steps, thereby suppressing competing reactions and catalyst poisoning. However, this approach also introduces a challenge: the activated intermediates may be separated in space, leading to kinetic barriers for C—N coupling and an increased likelihood of byproduct formation, if the two catalytic sites are spatially distant. Therefore, an optimal dual‐site design requires a uniform spatial distribution of the catalytic centers while ensuring that the activated species can effectively undergo C—N coupling.

#### 3.1.3 Heterostructural Electrocatalysts

Heterostructures, with tailored interfacial electronic interactions and synergistic active sites, have emerged as promising catalysts for urea synthesis. Their unique charge redistribution and multicomponent cooperation significantly enhance the activation of CO_2_ and nitrogenous molecules, and optimize C—N coupling efficiency, offering new opportunities for sustainable urea production.^[^ ^69^, ^70^ ^]^

**Figure 9:**
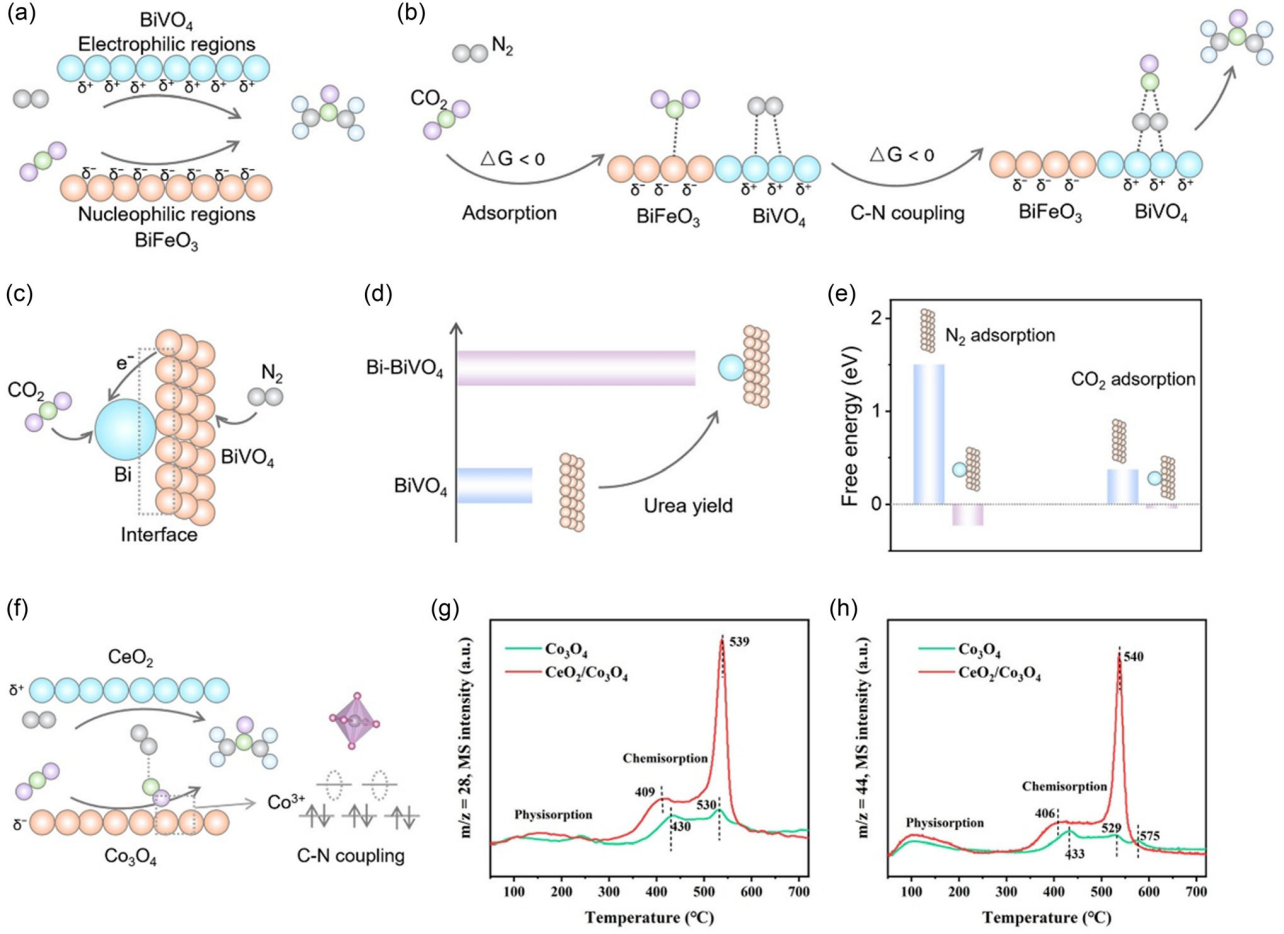
a) Structural diagrams for BiFeO_3_/BiVO_4_ heterojunction; b) free energy diagrams CO_2_ and N_2_ adsorption, and C—N coupling. Reproduced with permission.^[^ ^72^ ^]^ Copyright 2021, Royal Society of Chemistry. c) Schematic illustration of the charge transfer process in Bi–BiVO_4_; d) average urea yield rate of BiVO_4_ and Bi–BiVO_4_; e) CO_2_ and N_2_ adsorption energy on BiVO_4_ and Bi–BiVO_4_. Reproduced with permission.^[^ ^73^ ^]^ Copyright 2021, Wiley‐VCH. f) Schematic electrocatalytic urea production mechanism based on CeO_2_/Co_3_O_4_ p–n heterostructure synergistic effects; g) N_2_‐TPD and h) CO_2_‐TPD of Co_3_O_4_ and CeO_2_/Co_3_O_4_; Reproduced with permission.^[^ ^74^ ^]^ Copyright 2024, American Chemical Society.

The interface of heterostructures demonstrates advantages in both reactant adsorption and product selectivity. Multiheterojunction interfacial structure can efficiently suppress byproduct reactions forming H_2_, CO, N_2_, and NH_3_ in urea synthesis.^[^ ^71^ ^]^ The multiheterojunction interfacial architecture induced interfacial charge polarization through incomplete electron transfer at interface boundaries and multiple intermolecular interactions to optimize the intermediates’ adsorption/desorption abilities for suppressing byproducts. Unique interfacial effect also provides an optimized strategy for inert reactant activation and catalytic site poisoning. Yuan et al. developed a heterostructured BiFeO_3_/BiVO_4_ catalyst with a built‐in electric field, where interfacial contact between the two phases facilitates electron transfer from BiVO_4_ to BiFeO_3_, generating localized electrophilic regions on BiVO_4_ and nucleophilic domains on BiFeO_3_ surfaces.^[^ ^72^ ^]^ The local charge redistribution facilitated targeted adsorption and activation of inert N_2_ and CO_2_ molecules on the generated local electrophilic and nucleophilic regions (**Figure** **9**a,b), enabling the exothermic formation of the *NCON intermediate. The energy‐favorable C—N coupling pathway facilitated desorption of *N=N and *CO intermediates from active sites, which not only enhanced urea yield (4.94 mmol h^−1^ g^−1^ with a FE of 17.18% at −0.4 V vs RHE) but also mitigated catalyst poisoning through reduced *CO accumulation for improving the catalyst stability. The Mott–Schottky heterostructure can also induce localized charge redistribution, generating electrophilic and nucleophilic regions that synergistically adsorb and activate CO_2_ and N_2_ molecules. Hetero‐structural Bi–BiVO_4_ catalyst ensured spontaneous electron transfer from BiVO_4_ to Bi to form electrophilic region on BiVO_4_ and nucleophilic region on Bi (Figure 9c).^[^ ^73^ ^]^ The N_2_ and CO_2_ molecules can be targeted adsorbed on the BiVO_4_ and Bi surface by electrostatic interaction, respectively. The electrostatically synergistic adsorption effectively enhances the urea synthesis performance of Bi–BVO (Figure 9d). Furthermore, the adsorbed *N=N species‐mediated CO_2_‐to‐*CO conversion during CO_2_RR, which accelerated C—N coupling to yield the critical *NCON intermediate (Figure 9e). The formation of *NCON intermediates kinetically suppresses poisoning of catalytic sites by preemptively occupying reactive centers. The CeO_2_/Co_3_O_4_ p–n heterostructure exhibited the electrophilic and nucleophilic regions because of the built‐in electric field.^[^ ^74^ ^]^ However, in this p–n heterostructure, the CO_2_ targeted adsorption and activation was in the electrophilic region, and N_2_ targeted adsorption was in the nucleophilic region, which is different with the above examples (Figure 9f). This suggests that the adsorption and activation of CO_2_ and N_2_ at the nucleophilic and electrophilic regions on heterostructures’ surfaces may not be specific, and require a comprehensive consideration of the catalyst's surface chemistry and electronic properties. Upon this catalyst, the electrons in the *σ* orbitals of *N=N* intermediates can be easily accepted by the empty *e*_g_ orbitals of Co^3+^ in CeO_2_/Co_3_O_4_, which presented a low‐spin state (LS: t_2g_^6^ *e*_g_^0^), thus the N_2_ targeted adsorption was in the nucleophilic region. The N_2_ and CO_2_ temperature programmed desorption (TPD) results revealed that the CeO_2_/Co_3_O_4_ exhibited larger desorption peaks and higher desorption temperatures compared to Co_3_O_4_, indicating significantly enhanced adsorption capacity and binding energy for CO_2_ and N_2_ on the CeO_2_/Co_3_O_4_ surface (Figure 9g,h). This improvement can be attributed to the formation of electrophilic and nucleophilic regions in the CeO_2_/Co_3_O_4_ heterostructure, which strengthen the targeted adsorption of N_2_ and CO_2_. This strategic construction of electrophilic and nucleophilic regions offers mechanistic insights for adsorbing and activating inert molecules through targeted charge modulation for urea synthesis. Moreover, the 2D/2D heterostructures presented the local photophilic and electrophilic regions for targeted adsorption and activation of N_2_ and CO_2_.^[^ ^75^ ^]^ In this work, the Ru–Pd/WO_3_/MXene heterostructures have been reported, in which the metallic Ru−Pd can facilitate the formation of metal hydride and interfacial charge transfer between Ru–Pd/WO_3_/MXene. The WO_3_ adsorbed and activated N_2_ and 2D MXene offered higher electrical conductivity and adsorption sites for CO_2_ molecules activation. Indeed, the formation of both electrophilic/nucleophilic regions and photophilic/electrophilic regions is driven by interfacial charge transfer across the heterostructure. Both mechanisms utilize the affinity of these catalytic regions toward CO_2_ and N_2_ molecules to achieve adsorption and catalytic activation. Notably, these two types of regions share similarities in their design principles and catalytic functionalities.

This interfacial engineering strategy in heterostructures validates the structure–performance relationship, where the synergistic effects between electronic redistribution and structure assembly enhance the catalytic performance. By leveraging the characteristics of interfacial charge transfer in heterostructures, it is possible to engineer regions with distinct catalytic properties, thereby enhancing catalytic activity and specificity. This strategy holds significant potential for urea synthesis based on CO_2_RR and N/NO_*x*_RR as foundational catalytic processes.

### 3.2 Strategies for Enhancing ECUS Performance

#### 3.2.1 Catalysts Design

In the previous section, we summarized the reported electrocatalysts with potential applications in ECUS, including their primary catalytic centers and the mechanisms by which they activate reactant molecules. The catalytic performance is mainly regulated by catalyst designs, such as through the introduction of defect vacancies, surface modification, site design, selective facet engineering, or the construction of heterojunctions. These modification methods primarily aim to enhance catalytic performance by introducing more active sites or creating regions with higher affinity for reactant molecules on the catalyst surface. For example, introduction of defects on the catalyst surface can promote the adsorption of C‐reactants (or intermediates) and N‐reactants (or intermediates) to enhance the catalytic activity of the catalyst. Construction of O_v_ on low‐valent Cu‐supported TiO_2_ surfaces, O_v_ will simultaneously form on both Cu^δ+^ nanoparticle and TiO_2_ surfaces, where the Cu species preferred to adsorb CO_2_, while the O_v_ can enhance CO_2_ adsorption on the Cu^δ+^ species surface and promote NO_2_^−^ adsorption on the TiO_2_ surface.^[^ ^76^ ^]^ Moreover, O_v_ has been demonstrated to facilitate the desorption of intermediates (*CO and *NH_2_) and inhibit poisoning of catalytic sites, while simultaneously introducing lattice strain for catalysts to enhance C—N coupling.^[^ ^77^ ^]^ Surface modification can regulate catalytic performance by altering the surface chemistry and electronic properties of catalysts. Grafting vitamin B and F atoms onto carbon nanotube surfaces has been demonstrated to enhance the catalytic activity of carbon nanotubes.^[^ ^54^, ^78^ ^]^ F‐rich carbon nanotubes possessed more catalytically active surfaces compared to bare carbon nanotubes. The more electronegativity of F atoms relative to C atoms induced charge‐enriched high‐energy sites on the carbon nanotube surface, on which these sites exhibited superior catalytic activity compared to equivalent energy surfaces, facilitating the adsorption and catalytic conversion of C‐ and N‐reactants. The amino species generated from nitrogenous molecules can also facilitate the capture and activation of CO_2_.^[^ ^23^ ^]^ Introduction of amino species can effectively adsorb and activate CO_2_ molecules to overcome the inert of CO_2_. Furthermore, several specific facets or active phases of catalysts have demonstrated superior catalytic activity in urea synthesis. The Cu_2_O (100) facet exhibited higher urea activity and selectivity than the (110) facet and Cu (100) facet demonstrated higher activity than (110) and (111) facet, increasing (100) surface ratio on Cu_2_O and Cu can effectively enhance the catalytic activity in urea synthesis.^[^ ^18^, ^59^ ^]^

Introduction of dual catalytic sites on the catalyst surface to provide targeted adsorption of C‐ and N‐reactants is common modification for enhancing catalytic performance. The dual sites enable a targeted adsorption for reactants, separating the reactants, to provide tandem catalysis, which exhibits excellent performance than single‐atom catalysts in urea synthesis. The urea production of dual‐atom Pd_1_Cu_1_–TiO_2_ is near 6 times higher than that of single‐atom Pd_1_–TiO_2_.^[^ ^21^ ^]^ The experimental and density functional theory (DFT) results indicated that the dual‐atom Pd_1_Cu_1_ site is more favorable for producing urea, which showed a lower energy barrier for C—N coupling compared with Pd_1_ sites. Furthermore, the Ru–Cu dual sites also exhibited lower formation energy of the *COOH intermediate (rate‐determining step) than Cu sites.^[^ ^79^ ^]^ Catalysts with bimetallic catalytic sites can achieve higher selectivity and catalytic efficiency. Besides, introducing Lewis acid–base pairs or nucleophilic–electrophilic regions on the catalyst surface can also provide targeted adsorption and activity for C‐ and N‐containing reactants. For example, the construction of In/In–OH and Ni/Ni–OH Lewis acid–base pairs that utilize the electron donor–acceptor principle enabled selective adsorption of CO_2_ and N_2_, ensuring the effective activation process.^[^ ^67^, ^68^ ^]^ Fabricating heterojunction structures can enhance catalytic performance primarily through targeted adsorption of C‐ and N‐reactants. For example, the fabrication of p–n junctions and Mott–Schottky junctions leverages interfacial charge transfer characteristics at heterojunction interfaces to create nucleophilic‐electrophilic regions on catalyst surfaces, thereby promoting reactant adsorption and activation.^[^ ^73^, ^74^ ^]^ Furthermore, heterojunctions enable separated activation processes that enhance urea selectivity while suppressing byproduct formation.^[^ ^71^ ^]^

The design and modification of catalysts primarily aim to enhance surface affinity toward reactants and selectivity toward intermediates, with such modifications serving as critical pathways to boost catalytic efficiency.

#### 3.2.2 Catalytic System Design

**Figure 10:**
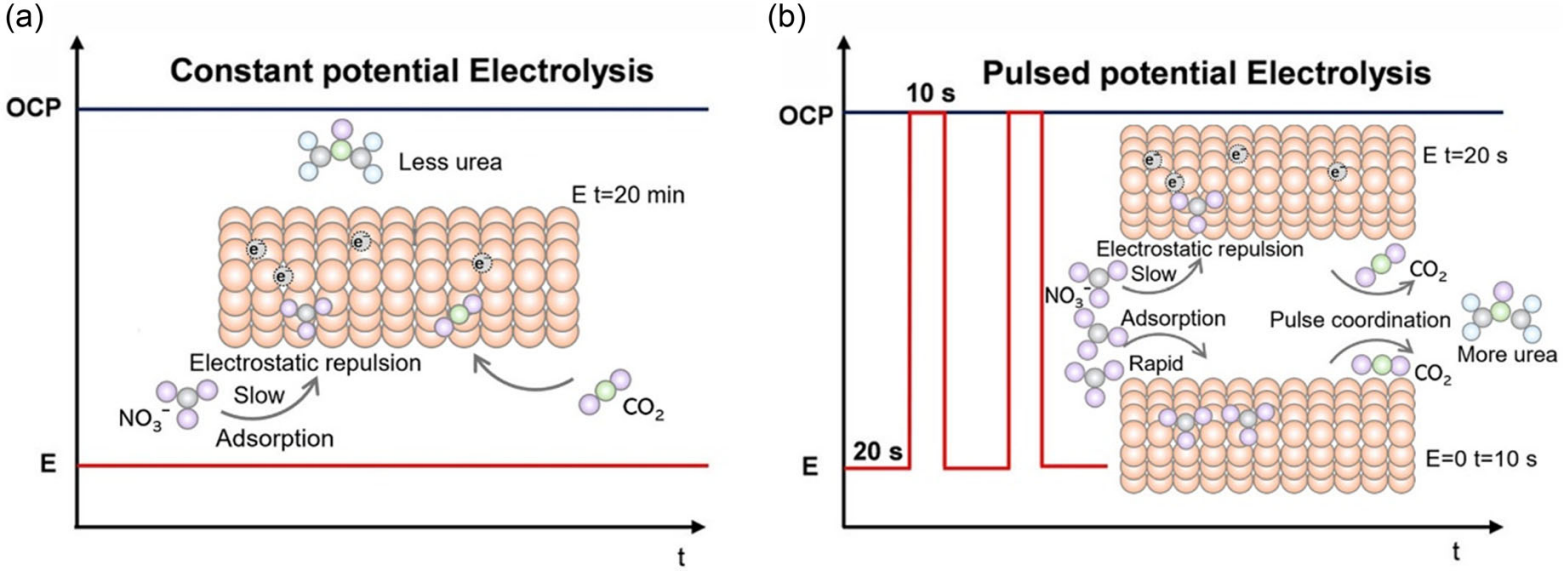
Schematic illustration of a) potentiostatic and b) pulsed electrolysis, the inset shows the corresponding reaction mechanism of potentiostatic or pulsed electrolysis. Reproduced with permission.^[^ ^25^ ^]^ Copyright 2024, Wiley‐VCH.

To enhance the efficiency and selectivity of urea synthesis, in addition to designing highly active catalysts, pretreatment of reactants or strategic design of the catalytic system can also effectively improve catalytic performance and product selectivity. Pulse‐assisted ECUS can effectively regulate the catalytic reaction at the catalyst‐electrolyte interface to enhance catalytic performance. Anionic group (NO_3_^−^/NO_2_^−^ as feedstock) is difficult to be adsorbed on the cathode surface due to the electrostatic repulsion, where a periodically alternated open circuit potential (OCP) was proposed to solve this issue. In a urea synthesis system with CO_2_ and NO_3_^−^ as feedstocks and CuSiO_*x*_ as catalyst, the introduction of OCP effectively promoted the adsorption of NO_3_^−^ on the Cu surface, overcame the hindrance from electrostatic forces, and enhanced the catalytic efficiency of interfacial active sites (**Figure** **10**a,b).^[^ ^25^ ^]^ For PdCu catalyst, the pulsed potentials between −0.2 and −0.8 V (vs. RHE) increased the local concentration of CO_2_/NO_3_^−^ and reduced the local pH for intermediate coupling.^[^ ^24^ ^]^ Stabilizing the pH on the electrode surface within a specific range is beneficial to maintain catalyst stability and suppresses the adverse effects of electrostatic repulsion on reactants, thereby ensuring long‐term operational stability. The local reaction environment, such as pH, concentration of reactants, around the electrode significantly influences catalytic activity. Pulsed potential can modify this reaction environment to regulate the performance and selectivity of ECUS.

To achieve high‐efficient catalytic system for urea synthesis, an appropriate electrolyte is also crucial. Typically, KOH and KNO_3_/KNO_2_ are commonly used electrolytes. However, the solubility of CO_2_ in these electrolytes is relatively low, making it challenging to achieve a high CO_2_ concentration at the sample surface, which limits CO_2_ participation in the reaction. This may lead to over‐catalysis of N‐reactants. By utilizing hybrid electrolytes to enhance CO_2_ concentration, such as KOH or KNO_3_/KNO_2_ combined with KHCO_3_ and acetonitrile, CO_2_ can be effectively driven to participate in the reaction, improving product selectivity. When acetonitrile was employed as the electrolyte, the stability of CO_2_^·−^ species on the catalyst surface was enhanced. This enabled CO_2_ to directly couple with NH_3_ in the form of CO_2_^·−^ instead of requiring activation into *COOH intermediates, significantly reducing the energy barrier of the reaction.^[^ ^80^ ^]^ Regulation of the local reaction environment via pulsed potentials and hybrid electrolytes not only facilitates reactant activation and coupling but also improves catalytic stability and selectivity. The relevant performances of ECUS are summarized in **Table** **1** for convenient comparison.

**Table 1:** Performance of ECUS.

	Electrocatalysts	Catalytic site	N source	Urea production	Potential	FE (%)	References
Single site	MoP	Mo	N_2_	12.4 mg g^−1^ h^−1^	−0.27 V	36.50	[37]
	Cu–TiO_2_	Ti^3+^	NO_2_^−^	20.8 μmol h^−1^	−0.4 V	43.10	[76]
	Bi‐riched GB	Bi	NO_3_^−^	4.6 mmol mg^−1^ h^−1^	−0.4 V	32.00	[94]
	6 Å–Cu	Cu	NO_3_^−^	7541.9 mg g^−1^ h^−1^	−0.41 V	51.97	[58]
	Co‐PMDA‐2‐mbIM	Co^4+^	N_2_	14.47 mmol g^−1^ h^−1^	−0.5 V	48.97	[95]
	Mo_2_C/C	Mo	NO_3_^−^	579.13 mg g^−1^ h^−1^	−0.5 V	44.80	[96]
	VB_12_‐CNTs/CP	Co	NO_3_^−^	164.04 μg h^−1^	−0.5 V	26.04	[78]
	In(OH)_3_–S	{100} facet	NO_3_^−^	533.1 mg g^−1^ h^−1^	−0.6 V	53.40	[60]
	F‐CNT‐300	C	NO_3_^−^	6.36 mmol g^−1^ h^−1^	−0.65 V	18.00	[54]
	Au NSs	Au	N_2_	86 mg g^−1^ h^−1^	−0.7 V	17.50	[86]
	TiN_0.3_	{100} facet	NO_3_^−^	1488 mg g^−1^ h^−1^	−0.72 V	43.10	[97]
	ZnO–O_v_	O_v_	NO_2_^−^	16.56 μmol h^−1^	−0.79 V	23.26	[55]
	Zn nanosheets	Zn	NO_3_^−^	39.3 mmol g^−1^ h^−1^	−0.8 V	31.80	[98]
	Cu‐GS‐800	Cu	NO_3_^−^	1800 mg g^−1^ h^−1^	−0.9 V	28.00	[43]
	Cu‐W_18_O_49_@ZIF‐8	Cu	N_2_	1.33 mmol g^−1^ h^−1^	−0.9 V	16.10	[99]
	Zn foil	Zn	NO	9.98 mmol g^−1^ h^−1^	−0.92 V	4.55	[100]
	Cu_2_O	{100} facet	NO_3_^−^	62.4 mmol g^−1^ h^−1^	−1.5 V	17.72	[59]
Dual sites	CuWO_4_	Cu; W	NO_3_^−^	98.5 mg g^−1^ h^−1^	−0.2 V	70.10	[65]
	ZnMn–N, Cl	Zn; Mn	N_2_	4 mmol g^−1^ h^−1^	−0.3 V	28.7	[101]
	Sb_*x*_Bi_1−*x*_O_*y*_	Sb; Bi	N_2_	307.97 mg g^−1^ h^−1^	−0.3 V	10.90	[102]
	Fe–N–C	Fe; Fe–N	N_2_	0.156 mmol g^−1^ h^−1^	−0.3 V	2.13	[103]
	Ru‐Cu_9_Bi/CNT	Cu; Bi	NO_3_^−^	40.0 mmol g^−1^ h^−1^	−0.4 V	75.60	[104]
	Mo‐PCN‐222(Co)	Mo; Co	NO_3_^−^	844.11 mg g^−1^ h^−1^	−0.4 V	33.90	[64]
	V_N_‐Cu_3_N‐300	Cu; Nv	N_2_	81 μg cm^−2^ h^−1^	−0.4 V	28.70	[105]
	CoN_3_‐CoAC/NC	Co; Co–N_3_	N_2_	20.83 mmol g^−1^ h^−1^	−0.4 V	23.73	[56]
	InOOH‐100	In; In–OH	N_2_	6.85 mmol g^−1^ h^−1^	−0.4 V	20.97	[68]
	Mo‐PdIn BNRs	Pd; In	NO_3_^−^	1016.49 mg g^−1^ h^−1^	−0.4 V	18.42	[106]
	Pd_1_Cu_1_/TiO_2_‐400	Cu; Pd	N_2_	3.36 mmol g^−1^ h^−1^	−0.4 V	8.92	[10]
	CuPd_1_Rh_1_‐DAA	Pd_1_–Cu; Rh_1_–Cu	NO_3_^−^	53.2 mmol g^−1^ h^−1^	−0.5 V	72.10	[107]
	Pd_4_Cu_1_‐FeNi(OH)_2_	Cu; Pd	NO_3_^−^	436.9 mmol g^−1^ h^−1^	−0.5 V	66.40	[108]
	PdCu/CBC	Pd; Cu	NO_3_^−^	763.8 mg g^−1^ h^−1^	−0.5 V	59.70	[109]
	H‐PdZn	Pd; Zn	NO_3_^−^	314.17 mg g^−1^ h^−1^	−0.5 V	24.39	[110]
	Ni_3_(BO_3_)_2_‐150	Ni; Ni^2+^	N_2_	9.70 mmol g^−1^ h^−1^	−0.5 V	20.36	[67]
	XC72R‐AuPd	Au; Pd	NO_3_^−^	204.2 mg g^−1^ h^−1^	−0.5 V	15.60	[111]
	Pt_1_Cu_1_–TiO_2_	Pt; Cu	N_2_	51.71 mol mol_Pt+Cu_^−1^ h^−1^	−0.5 V	11.53	[112]
	CuSiO_*x*_	Cu–O–Si; Si	NO_3_^−^	1606.1 mg g^−1^ h^−1^	−0.6 V	79.01	[25]
	FeNC–Fe_1_N_4_ /C	Fe; Fe–N	NO_3_^−^	30.3 mmol g^−1^ h^−1^	−0.6 V	66.50	[66]
	Vo‐S‐IO‐6	O_v_; {100} facet	NO_3_^−^	910.4 mg g^−1^ h^−1^	−0.6 V	60.6	[61]
	CoPc‐COF@TiO_2_ NTs	Co; Ti	NO_3_^−^	1205 μg cm^−2^ h^−1^	−0.6 V	49.00	[113]
	Cu–MoSe_2_	Cu; Mo	NO_3_^−^	1235 mg g^−1^ h^−1^	−0.6 V	23.43	[114]
	CuPc NTs	N; Cu	N_2_	143.47 mg g^−1^ h^−1^	−0.6 V	12.99	[115]
	Fe(a)@C‐Fe_3_O_4_/CNTs	Fe^0^; Fe^δ+^	NO_3_^−^	1341.3 mg g^−1^ h^−1^	−0.65 V	16.50	[116]
	Cu_99_Ni_1_	Cu; Ni	NO_2_^−^	655.4 μg cm^−2^ h^−1^	−0.7 V	39.80	[117]
	SrCo_0.39_Ru_0.61_O_3−δ_	Co; Ru	NO_3_^−^	1522 mg g^−1^ h^−1^	−0.7 V	34.10	[77]
	CoPc‐MoS_2_	Co; Mo	N_2_	175.6 mg g^−1^ h^−1^	−0.7 V	15.12	[62]
	FeNi/NC	Fe; Ni	NO_3_^−^	496.5 mg g^−1^ h^−1^	−0.9 V	16.58	[118]
	Cu_1_Au_8_@CeO_2_	Cu; Au	NO_3_^−^	813.6 mg g^−1^ h^−1^	−0.94 V	45.20	[119]
	Cu@Zn	Zn; Cu	NO_3_^−^	7.29 μmol cm^−2^ h^−1^	−1.02 V	9.28	[120]
Heterostructure	CeO_2_/Co_3_O_4_	CeO_2_; Co_3_O_4_	N_2_	5.81 mmol g^−1^ h^−1^	−0.2 V	30.05	[74]
	BiFeO_3_/BiVO_4_	BiFeO_3_; BiVO_4_	N_2_	4.94 mmol g^−1^ h^−1^	−0.4 V	17.18	[72]
	Sn/Cu_2_O‐α	Sn; Cu_2_O‐α	NO_3_^−^	32.35 μmol cm^−2^ h^−1^	−0.4 V	13.50	[121]
	Bi–BiVO_4_	Bi; BiVO_4_	N_2_	5.91 mmol g^−1^ h^−1^	−0.4 V	12.55	[73]
	Ag–CuNi(OH)_2_	Ag(111); CuNi(OH)_2_	NO_3_^−^	25.6 mmol g^−1^ h^−1^	−0.5 V	46.10	[122]
	Bi_2_S_3_/N‐RGO	Bi_2_S_3_; N‐RGO	N_2_	4.4 mmol g^−1^ h^−1^	−0.5 V	7.50	[123]
	Ru–Pd/WO_3_/MXene	Ru; Pd	N_2_	227 mg g^−1^ h^−1^	−0.6 V	23.70	[75]
	O–Bi_M_/CuO_*X*_	O–Bi; Bi–Cu	NO_3_^−^	2180.3 mg g^−1^ h^−1^	−0.6 V	23.50	[124]
	Co–NiO_*x*_@GDY	Co–NiO_*x*_	NO_2_^−^	913.2 mg g^−1^ h^−1^	−0.7 V	64.30	[71]
	Fe^II^–Fe^III^OOH@BiVO_4_	Fe^II^–Fe^III^OOH; BiVO_4_	NO_3_^−^	13.8 mmol h^−1^	−0.8 V	11.50	[70]

## 4 Photocatalytic Urea Synthesis

### 4.1 Photocatalysts

The PCUS was proposed by Hubbard et al. from CO and NH_3_ in 1975.^[^ ^8^ ^]^ Afterward, Kuwabata et al. employed the size‐quantized TiO_2_ nanocrystals as photocatalysts to successfully convert CO_2_ and NO_3_^−^ into urea in CO_2_‐saturated propylene carbonate solution containing 20 mmol dm^−3^ LiNO_3_ and 1 mol dm^−3^ 2‐propanol (hole scavenger) in 1998.^[^ ^9^ ^]^ In addition to urea, this reaction also produced some byproducts, such as methanol (produced at the beginning of the illumination), acetone (produced in the beginning of the illumination), NH_4_^+^ (produced after illumination for ≈3 h), and H_2_, from Equation (11) to (15).

(11) $$2{\text{NO}}_{3}^{-}+{\text{CO}}_{2}+18H^{+}+16e^{-}\to {\left({\text{NH}}_{2}\right)}_{2}C=O+7H_{2}O$$

(12) $${\text{CH}}_{3}\text{CH}\left(\text{OH}\right){\text{CH}}_{3}\to {\text{CH}}_{3}{\text{COCH}}_{3}+2H^{+}+2e^{-}$$

(13) $${\text{CO}}_{2}+6H^{+}+6e^{-}\to {\text{CH}}_{3}\text{OH}+H_{2}O$$

(14) $${\text{NO}}_{3}^{-}+10H^{+}+8e^{-}\to {\text{NH}}_{4}^{+}+3H_{2}O$$

(15) $$2H^{+}+2e^{-}\to H_{2}$$

The PCUS primarily relies on excited photogenerated electrons to catalyze CO_2_ and nitrogenous small molecules, where the energy carried by these excited‐state electrons influences their catalytic capability. The energy of photogenerated electrons is mainly related to the CB position and bandgap width of semiconductor photocatalysts. There are no specific catalytic potential requirements established in the photocatalytic field for urea synthesis. In general, semiconductor photocatalysts with wider bandgaps and higher CB position can exhibit stronger catalytic capabilities for CO_2_RR and N/NO_*x*_RR. However, the wider bandgaps will weaken light absorption and photon utilization efficiency, which limits the solar‐to‐chemical energy conversion efficiency. Therefore, the design of photocatalysts should consider not only sufficient reduction potentials to activate CO_2_ and nitrogenous small molecules, but also the light absorption range of photocatalysts. This creates a critical trade‐off that requires careful consideration. Furthermore, the energy of photogenerated electrons lacks a fixed range, in contrast to electrocatalysis, where the applied external voltage enables precise control. This makes it challenging to regulate the catalytic potential, resulting in relatively low product selectivity. Therefore, it is difficult to improve product selectivity through the tuning of CB and valence band (VB) positions in photocatalysts. Enhancing product selectivity may be more effectively achieved by engineering surface catalytic sites on photocatalysts to promote the targeted adsorption of reactants or stabilize specific intermediates. The reported semiconductor photocatalysts to date are mainly based on TiO_2_, CeO_2_, WO_3_, CdS, FeS, CoWO_4_, SrTiO_3_, Cs_2_CuBr_4_, and SiW_6_Mo_6_, with key catalytic sites, such as Pt, Ru, Cu, Pd, Mo, W, Ti, Fe, Mn, and O_v_, playing a crucial role in the reaction.

**Figure 11:**
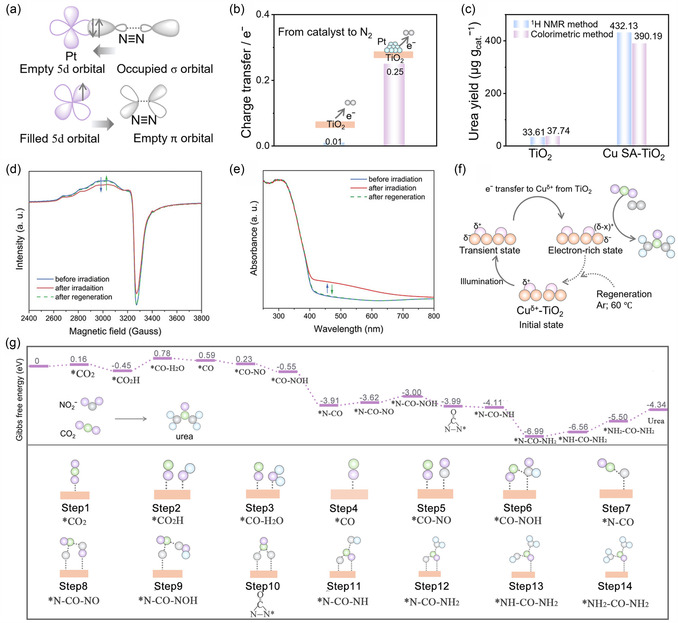
a) A simplified schematic diagram of N_2_ bonding to a Pt center; b) Bader calculation of N_2_ on TiO_2_ and Pt cluster/TiO_2_. Reproduced with permission.^[^ ^81^ ^]^ Copyright 2024, Wiley‐VCH. c) The urea yield of pure TiO_2_ and Cu SA–TiO_2_ detected by colorimetric and ^1^H‐NMR methods at 365 nm monochromatic light (433 mW cm^−2^) after 1 h; d) electron paramagnetic resonance and e) UV–vis diffuse reflectance spectra of Cu SA–TiO_2_ during an irradiation and regeneration cycle process; f) proposed mechanism of reversible and cooperative photocatalysis in Cu SA–TiO_2_. Reproduced with permission.^[^ ^83^ ^]^ Copyright 2022, Wiley‐VCH. g) DFT calculations for the PCUS process through nitrate and CO_2_ coreduction. Reproduced with permission.^[^ ^84^ ^]^ Copyright 2024, Wiley‐VCH.

The primary challenge lies in the activation of inert CO_2_ and nitrogenous molecules in the urea synthesis, especially linearly symmetric and nonpolar CO_2_ and N_2_ molecules. The Pt cluster/TiO_2_ photocatalyst offers a reference to activate the N_2_. The empty 5*d* orbitals of Pt sites on Pt cluster demonstrated affinity with N_2_ via σ–π* interaction.^[^ ^81^ ^]^ The empty 5*d* orbitals initially accepted electrons from the occupied *s* orbitals of N_2_. Then, electron‐enriched 5*d* orbitals transferred electrons to the vacant π* orbitals of N_2_ to form σ–π* interaction for effective activation of N_2_ molecule (**Figure** **11**a,b). The electron transfer between bonding orbitals can enhance the adsorption and activation between catalytic sites and reactant molecules. The N_2_ molecules that received electrons are ultimately catalyzed into *NH–NH intermediates, which then undergo C—N coupling. The regulation of catalyst surface properties also facilitates the promotion of CO_2_ and N_2_ adsorption and activation. The introduction of O_v_ on CeO_2_ induces electronic defects, corresponding to the formation of two Ce^3+^ cations that generate polarons.^[^ ^82^ ^]^ Polaron‐rich surfaces exhibited dual strong adsorption capabilities toward both the electron‐rich N atom in N_2_ and the electron‐deficient C atom in CO_2_, which can easily realize targeted adsorption on the active site, respectively. This site‐specific adsorption arises from the electrostatic interactions between polaronic regions and target atoms, enabling precise activation of both reactants at designated active sites. This site‐specific interaction is reflected in the calculated adsorption energies of −0.24 eV for N_2_ and −0.27 eV for CO_2_. The scarcity of catalytically active photogenerated electrons is also a critical factor limiting catalytic activity. Semiconductor catalysts must continuously transfer photogenerated electrons from their surface to reactant molecules to attain sufficient catalytic capacity for activating inert gas molecules. The reversible Cu single atom, serving as an electron transfer bridge anchored on the TiO_2_ surface, provides a driving force that accelerates the electron transfer from the TiO_2_ surface to N_2_ and CO_2_.^[^ ^83^ ^]^ The multivalent Cu species played a pivotal role to enhance the urea production (Figure 11c). Upon the generation of photogenerated electrons in TiO_2_, Cu first acted as an electron acceptor to capture and store photogenerated electrons, leading to valence state decrease. Subsequently, Cu served as an electron donor to transfer electrons to reactant molecules, accompanied by its valence state increase. The valence state of Cu dynamically oscillated between 0 and +2 in photocatalytic process, realizing the stabilization of catalytic species and enabling a sustained electron supply (Figure 11d–f). The electron extraction rate from TiO_2_‐to‐Cu‐to‐CO_2_/N_2_ is over 30 times faster than the TiO_2_‐to‐CO_2_/N_2_, which provided the assurance of abundant and continual photogenerated electrons to drive the multielectron‐demanding cophotoactivation of N_2_ and CO_2_, thereby achieving a remarkable urea production. A Ru–TiO_2_ photocatalyst consisted of Ru single atoms supported on TiO_2_ to provide “electronic pump” for N_2_ activation.^[^ ^84^ ^]^ These Ru atoms continuously transferred photogenerated electrons from the TiO_2_ surface to N_2_ molecules, driving the catalytic process. This mechanism prevents the accumulation of photogenerated electrons on the TiO_2_ surface to avoid an electronegativity effect and electron loss. Continuous input of photogenerated electrons enhances catalytic activity of photocatalysts by facilitating charge accumulation. Ultimately, N_2_ is activated into the *NNOH intermediate, which then undergoes C—N coupling with the *CO intermediate formed from CO_2_ to produce urea (Figure 11g). Niu et al. constructed a heterojunction catalyst Pd(111)/CeO_2_(111), leveraging interfacial charge transfer to establish a space‐charge region at the heterointerface.^[^ ^85^ ^]^ This engineered interface demonstrated exceptional dual activation capabilities for both N_2_ and CO_2_. Specifically, the simultaneous generation of *N=N and *CO active species at the heterojunction promoted the formation of the *NCON intermediate while effectively suppressing the competing pathway via *NNH intermediates. The above selectivity modulation significantly reduced NH_*x*_‐related byproducts, thereby enhancing urea yield.

The coactivation of CO_2_ and nitrogenous molecules is fundamental to PCUS. Effective regulation of the photocatalyst's activation capability is essential, as excessively strong or weak activation can hinder the reaction. This necessitates the selective activation of reactant molecules into specific intermediates that facilitate C—N coupling. These activation processes are controlled by well‐defined catalytic sites on the catalyst surface. Therefore, a comprehensive understanding of these catalytic sites is crucial for the rational design of catalysts and the optimization of reaction pathways.

### 4.2 Strategies for Enhancing PCUS Performance

#### 4.2.1 Catalysts Design

In photocatalysis, the energy source is mainly from the photogenerated electrons. To activate inert reactant molecules, the design of photocatalysts focuses on light absorption enhancement, improvement of the adsorption capacity for reactants and sufficient photogenerated electrons to drive reactant activation. Surface modifications, such as the design of metal catalytic centers and the introduction of defects, can enhance light absorption, promote efficient separation of photogenerated carriers, and facilitate the adsorption and activation of reactants. The surface plasmonic resonance effect of noble metals, such as Au and Ag, can significantly enhance the light‐harvesting capability of semiconductors, while the generated hot carriers contribute to catalytic activity.^[^ ^86^ ^]^ Thus, incorporating these noble metals onto photocatalyst surfaces and tailoring their structure and size to harness plasmon‐assisted photocatalysis is an effective strategy. In addition, these metal catalytic centers can act as electron‐rich sites, continuously supplying energy for the catalytic reactions. Ru atoms loaded on the surface of TiO_2_ have been employed as “electron pumps” to prevent the recombination of transferred electrons on the catalyst surface and the formation of an electronegative shielding layer.^[^ ^84^ ^]^ This facilitates the transfer of electrons from N_2_ to the TiO_2_ support, effectively activating N_2_. Pd and Pt clusters also provide favorable sites for electron accumulation and N_2_ activation. Pd(111) exhibited targeted adsorption of N_2_ molecules, which stabilized the C—N coupling intermediate (*NCON) and suppressed the formation of the competing *NNH intermediate.^[^ ^82^ ^]^ This inhibition of N‐related byproducts enhanced the selectivity toward urea. Introduction of O_v_ can trigger local reconstruction on the photocatalyst surface to modulate the surface electron cloud distribution, which contributes to improved charge separation efficiency and facilitates the adsorption and activation of reactant molecules. Constructing O_v_ within TiO_*x*_ shell provided a pathway for electron transfer from the TiO_*x*_ to the CO_2_/NO_3_^−^, suppressing the recombination of photogenerated charge carriers.^[^ ^87^ ^]^ Moreover, the regulation of electron distribution between CO_2_/NO_3_^−^ and the TiO_*x*_ surface by O_v_ effectively promoted the adsorption and activation of CO_2_ and NO_3_^−^, while also facilitating the rate‐determining step of OCONH_2_ → HOCONH_2_. Constructing heterojunctions is also an effective strategy to enhance photocatalytic performance by enabling engineered interfaces and spatially separated dual catalytic regions. The FeS in SrTiO_3_–FeS–CoWO_4_ Z‐scheme heterostructure effectively modulates the interfacial properties between SrTiO_3_ and CoWO_4_, accelerated charge transfer, and provide dual catalytic regions for CO_2_ and N_2_ adsorption.^[^ ^88^ ^]^

The design of photocatalysts primarily focuses on surface modifications and construction of heterojunctions. These strategies aim to introduce catalytic centers that facilitate light harvesting, promote the separation of photogenerated charge carriers, enhance reactant adsorption, or regulate the stability of C—N coupling intermediates, for improving catalytic efficiency and selectivity.

#### 4.2.2 Catalytic System Design

**Figure 12:**
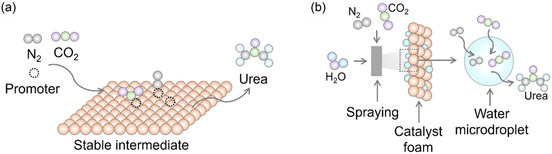
a) Scheme digamma of “catalytic promoter” in urea synthesis. b) Schematic illustration of microdroplet catalytic reaction. Reproduced with permission.^[^ ^90^ ^]^ Copyright 2023, American Chemical Society.

The PCUS system mainly comprises the reactants, catalysts, reaction solution, reaction cell, and light source. At present, efforts concerning the reaction system are primarily directed toward the pretreatment of reactant molecules and the reaction solution. The use of trace additives can activate reactants to achieve higher catalytic activity and selectivity (**Figure** **12**a). The introduction of trace amounts of NO as “catalytic promoter” into a reaction system with N_2_ and CO_2_ as reactants resulted in a nearly tenfold enhancement in urea production compared to only N_2_ and CO_2_ system.^[^ ^89^ ^]^ NO promoted the formation of NH_4_^+^ in the system and shifted the reaction equilibrium during CO_2_ activation (CO_2_ + H_2_ ⇌ HCOOH ⇌ CO + H_2_O), thereby facilitating the generation of HCOOH. This enhanced formation of COOH and NH_4_^+^ promotes their coupling (NH_4_^+ −^O—C(=O)–NH_2_ → CO(NH_2_)_2_), ultimately boosting urea synthesis. The use of such “catalytic promoters” to regulate the equilibrium of reversible reactions or enhance the reactivity of inert molecules serves as an effective strategy to improve catalytic performance. However, “catalytic promoters” that do not participate in the reaction or are difficult to separate may compromise product selectivity. The amino species generated from nitrogenous molecules can also facilitate the capture and activation of CO_2_. Designing tandem photocatalysts that preferentially activate N‐reactants and subsequently utilize the generated amino species to adsorb and activate CO_2_ molecules can effectively address the aforementioned issue. To achieve high performance in PCUS, sufficient contact between the photocatalysts and reactant molecules is crucial. In the PCUS system, KNO_3_, KNO_2_, and H_2_O are typically used as the reaction solution, but CO_2_/N_2_ molecule exhibits extremely low solubility in aqueous solution. To address this issue, a nebulizing gas method was proposed. Microdroplets containing water, CO_2_, and N_2_ were sprayed onto a CuBi_2_O_4_‐coated graphite mesh, and were proposed to enhance the contact between the reactants and the catalyst to improve the catalytic efficiency.^[^ ^90^ ^]^ By forming microdroplets, essential C, N, and H sources for urea synthesis were provided, with each microdroplet acting as an individual reaction cell (Figure 12b). The atomization device ensured a continuous supply of reactants, while the subsequent separation process efficiently isolated the produced urea. This innovative catalytic system not only addresses the issue of reactant depletion in later reaction stages but also offers a scalable framework for industrial production. The relevant performances of PCUS are summarized in **Table** **2** for convenient comparison.

**Table 2:** Performance of PCUS.

Photocatalysts	N source	Light source	Urea production	Selectivity	References
Cs_2_CuBr_4_/TiO_*x*_–Ar	NO_3_^−^	LED lamp (95.34 mW cm^−2^)	∼0.17 μg h^−1^ L^−1^	88.45%	[87]
Ru–TiO_2_	N_2_	420 nm monochromatic light	24.95 μmol h^−1^ g_cat._^−1^	–	[84]
Cu SA–TiO_2_	N_2_	365 nm monochromatic light	432.12 μg g_cat._^−1^	–	[83]
CeO_2_‐500	N_2_	300 W Xe lamp	15.5 μg h^−1^	–	[82]
SrTiO_3_–FeS–CoWO_4_	N_2_	300 W Xe lamp with 420 nm filter	8054.2 μg h^−1^ g_cat._^−1^	–	[88]
Ni_1_–CdS/WO_3_	N_2_	300 W Xe lamp with 385 nm filter	78 μM h^−1^	–	[125]
Pd–CeO_2_	N_2_	300 W Xe lamp	9.2 μmol h^−1^ g^−1^	–	[85]
SiW_6_Mo_6_@MIL‐101(Cr)	N_2_	300 W Xe lamp	1148 mg h^−1^ g_cat._^−1^	100%	[126]
Ti^3+^–TiO_2_/Fe–CNTs	N_2_	300 W Hg lamp	710.1 μmol L^−1^ g_cat._^−1^	–	[127]
NCP/ZIS	N_2_	300 W Xe lamp	19.6 μmol g^−1^ h^−1^	–	[128]

## 5 Conclusion and Outlook

**Figure 13:**
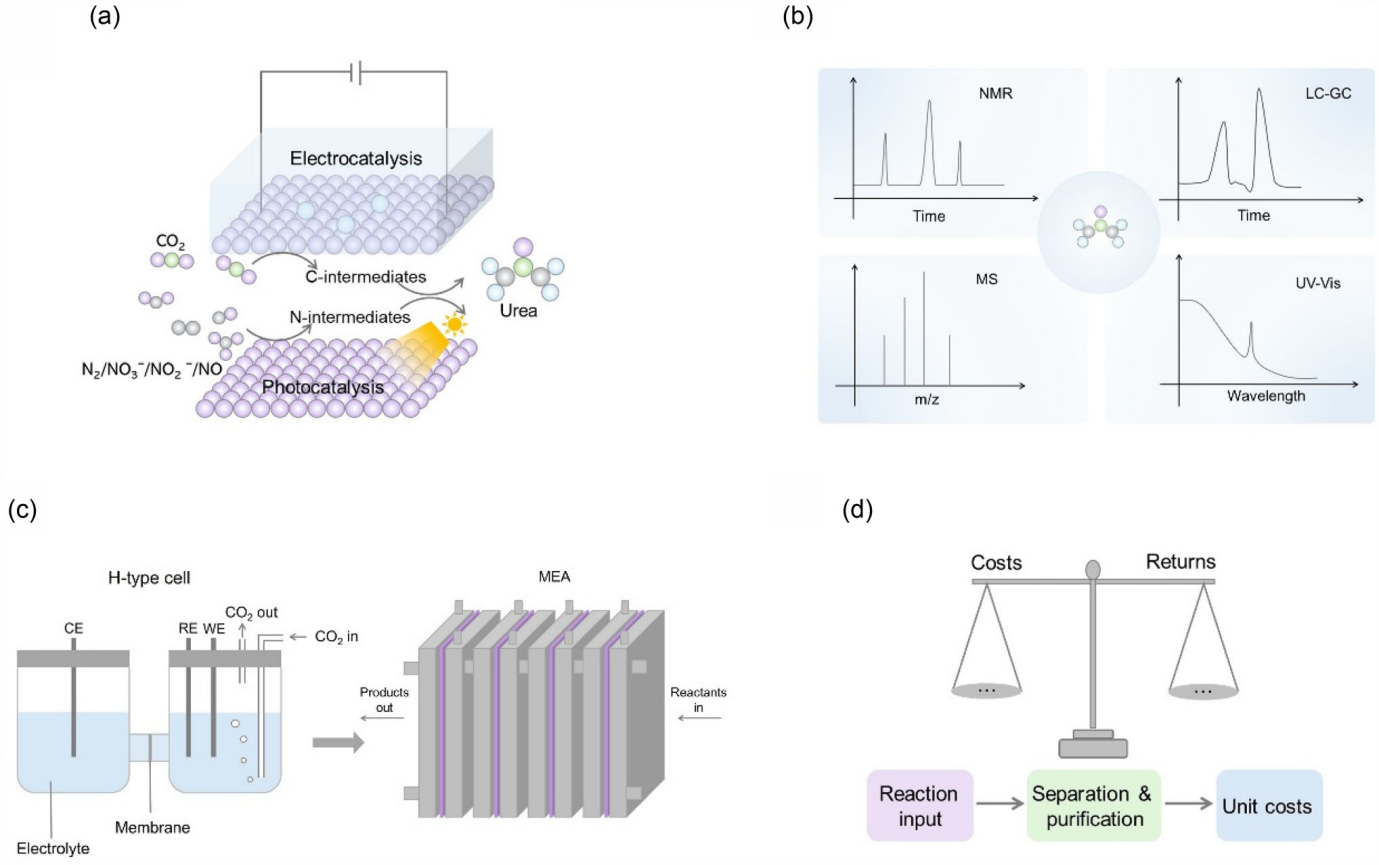
Overview of future perspectives for urea production, including: a) catalytic system design, b) standardization of product analysis methods, c) scale‐up pathways from laboratory to industrial application, and d) techno‐economic analysis.

In this review, we systematically summarized the fundamental reaction mechanisms and key challenges in ECUS/PCUS system, urea detection methods, state‐of‐the‐art catalytic systems and their performance metrics, and innovative strategies to enhance catalytic activity through catalysts engineering and process optimization. The N‐feedstocks are N_2_, NO_3_^−^, NO_2_^−^, and NO with different dissociation energy between these feedstocks, N≡N (941 kJ mol^−1^) > C=O (750 kJ mol^−1^) > N=O (204 kJ mol^−1^). There are different C—N coupling mechanisms, one‐step coupling for N_2_ and CO_2_; two‐step for NO_3_^−^/NO_2_^−^/NO and CO_2_. Building upon the mechanistic understanding of these catalytic processes, the development of high‐performance catalysts and catalytic systems, coupled with standardized performance evaluation criteria, is imperative. Furthermore, the most critical and worthwhile step in urea synthesis lies in its scale‐up from laboratory to industrial production, where reactor design optimization and techno‐economic analysis become paramount. Therefore, future advancements in this field should focus on the following key aspects (**Figure** **13**):

1) Design of an efficient catalytic system that encompasses both catalysts and reaction system. The urea synthesis reaction utilizes CO_2_ and N_2_ as feedstocks, requiring these gases to exhibit good solubility in the electrolyte or solution. Since most aqueous solutions currently struggle to provide adequate solubility, it becomes necessary to introduce “cosolvents” such as KHCO_3_ or acetonitrile, or employ specific ions (e.g., Li^+^) as “carriers” to transport these gases. Furthermore, pretreatment of reactants (e.g., plasma treatment and addition of “promoters”) or premixing reactants and solvents to form microdroplets that serve as isolated catalytic cells can effectively boost the reactivity of inert molecules to promote catalytic efficiency. Urea synthesis demands catalysts with dual‐functional activation capabilities to simultaneously activate CO_2_ and nitrogenous small molecules. Designing tandem catalysts or catalytic systems to realize stepwise catalysis is recommended. For example, some ferroelectric catalysts possess switchable polarization under light illumination and exhibit distinct catalytic properties before and after polarization reversal. Ju et al. reported that ferroelectric phase of AgBiP_2_Se_6_ showed higher oxidizability, while paraelectric phase possessed stronger reducibility.^[^ ^91^ ^]^ Inspired by it, the switchable feature holds promise for the stepwise activation of CO_2_ and nitrogenous small molecules by electro/photo‐switchable catalytic system. For instance, a switchable ferroelectric catalyst can be designed to activate CO_2_ via electrocatalysis, and subsequently reverse its polarization upon light excitation to enable effective activation of nitrogenous molecules. During the reaction, light switching should be applied intermittently to facilitate CO_2_ and nitrogenous molecules activation, similar to pulsed potentials. Catalysts with light‐induced polarization switching or tunable functionalities are worth exploring for such systems.

2) Standardization of product analysis and activity reporting protocols. At the laboratory stage, urea yield remains at low levels. Minor experimental errors during product detection can lead to significant inaccuracies. For such reactions, standardized operating procedures are essential to ensure scientifically valid conditions. It is crucial to develop a standardized protocol to describe key parameters such as current density, product concentration, and conversion efficiency. Furthermore, current urea detection primarily relies on colorimetric methods, chromatography, and mass spectrometry. While colorimetric methods are operationally convenient, factors such as color development time and interference from N‐containing compounds can severely affect results. Therefore, it is recommended to employ chromatography or mass spectrometry combined with isotopic analysis. In ECUS system, key parameters include FE, production rate, and yield. When evaluating production rate, it is essential to consider catalyst loading, electrode geometric area, and electrochemically active surface area. During electrode preparation, factors such as catalyst loading, coating thickness, and catalyst deposition methods significantly influence performance. Consequently, it is advised to report productivity via followed formula: urea yield (mol or mg)/[electrochemically active surface area (cm_ECSA_^−2^) Í time (h)]. In PCUS system, light type and intensity, reaction temperature, and sacrificial agent or cocatalyst should be described. The reaction temperature should be clearly reported in photocatalysis to assess the potential thermal effects. However, it is often overlooked. Additionally, the impact of light on urea decomposition should also be evaluated. Visible light, abundant at the Earth's surface, is recommended as the preferred excitation source. For productivity metrics, the standard reporting format should be reported in terms of urea yield (mol or mg)/[catalysts quality (g or mg) Í time (h)].

3) The transition from laboratory to industrial scale represents is the most critical step in urea synthesis, requiring scalable reactors for experimental validation. While traditional H‐cells are suitable for catalyst screening and mechanistic studies in laboratory settings, they suffer from inherent limitations for scale‐up, such as low mass transfer efficiency, poor gas–liquid–solid contact, and difficulty in maintaining steady‐state reaction conditions. These limitations hinder their applicability in continuous production scenarios. In contrast, practical applications necessitate the adoption of more advanced reactor configurations such as flow cells, membrane electrode assemblies (MEAs), and gas diffusion electrode systems. These continuous‐flow systems offer enhanced mass and electron transport, improved reactant accessibility to active sites, and better control over reaction parameters. In addition, several key performance metrics should also be achieved to approach industrially relevant reaction rates and selectivity. For example, in electrocatalytic system, operating current density ≥ 100 mA cm^−2^; FE (urea) ≥ 50%; energy efficiency of 40–50%; operational stability  ≥ 1000 h; feedstock sources such as industrial CO_2_ and nitrogen species (NO_3_^−^, NO_2_^−^, and NO) derived from flue gas or wastewater. In photocatalytic system, apparent quantum efficiency: 5–10%; selectivity ≥ 60%; catalyst lifetime ≥ 1000 h; light source: sunlight; reactor design: photoflow reactors or light‐guiding systems.

4) Techno‐economic analysis. Currently, economic feasibility analysis for ECUS and PCUS is underdeveloped. However, such analysis is essential for scale‐up from laboratory research to practical production. Key factors such as capital expenditure, operating costs, and the complexity of product separation and purification should be evaluated to estimate the overall process cost.^[^ ^92^ ^]^ Additionally, the yield, selectivity, and production cost of urea, along with the potential benefits from wastewater (NO_3_^−^/NO_2_^−^) and exhaust gas (CO_2_ and NO) treatment, should be analyzed to comprehensively assess the techno‐economic feasibility of the reaction.^[^ ^93^ ^]^ In an electrocatalytic system using a MEA with an anion exchange membrane (AEM), 1 M KOH, NO_3_^−^, and CO_2_ as feedstocks, the capital expenditure primarily includes the cost of the membrane and balance‐of‐plant components. The operating costs encompass the expenses of cathode and anode catalysts, electricity, electrolyte, and system maintenance. Therefore, a comprehensive techno‐economic analysis requires data on the prices of cathode and anode catalysts, AEM, and KOH, as well as key performance indicators such as current density, cell voltage, product selectivity, urea separation and purification costs, maintenance costs, the market price of urea, and the potential benefits from NO_3_^−^ and CO_2_ treatment. In a photocatalytic system using a photoflow reactor equipped with a carbon cloth‐supported catalyst, with NO_3_^−^ and CO_2_ as feedstocks and sunlight as the light source, several factors must be considered for a comprehensive techno‐economic analysis. These include the cost of quartz glass plates, the price of carbon cloth and photocatalysts, the costs associated with urea separation and purification, the market price of urea, urea selectivity, as well as the potential revenue from the treatment of NO_3_^−^‐containing wastewater and CO_2_ emissions. The economic framework developed based on these key parameters can serve as a guide for future research.

## Conflict of Interest

The authors declare no conflict of interest.

## Author Contributions

**Lizhen Liu**: writing—original draft (lead); writing—review & editing (lead). **Lin Zhou**: writing—original draft (equal). **Longcheng Zhang**: writing—review & editing (supporting). **Hongwei Huang**: writing—review & editing (supporting). **Xin Zhao**: writing—review & editing (equal). **Zhichuan J. Xu**: writing—review & editing (lead).
